# Magnetic resonance elastography resolving all gross anatomical segments of the kidney during controlled hydration

**DOI:** 10.3389/fphys.2024.1327407

**Published:** 2024-02-07

**Authors:** Marcos Wolf, Omar Darwish, Radhouene Neji, Michael Eder, Gere Sunder-Plassmann, Gertraud Heinz, Simon Daniel Robinson, Albrecht Ingo Schmid, Ewald V. Moser, Ralph Sinkus, Martin Meyerspeer

**Affiliations:** ^1^ High Field MR Center, Center for Medical Physics and Biomedical Engineering, Medical University of Vienna, Vienna, Austria; ^2^ School of Biomedical Engineering and Imaging Sciences, King’s College London, London, United Kingdom; ^3^ MR Research Collaborations, Siemens Healthcare Limited, Frimley, United Kingdom; ^4^ Department of Medicine III, Division of Nephrology and Dialysis, General Hospital and Medical University of Vienna, Vienna, Austria; ^5^ Institut für Diagnostische und Interventionelle Radiologie, Universitätsklinikum St. Pölten, Sankt Pölten, Austria; ^6^ High Field MR Centre, Department of Biomedical Imaging and Image-Guided Therapy, Medical University of Vienna, Vienna, Austria; ^7^ Centre of Advanced Imaging, University of Queensland, Brisbane, QLD, Australia; ^8^ Institut National de La Santé et de La Recherche Médicale, U1148, Laboratory for Vascular Translational Science, Paris, France

**Keywords:** MRE, quantitative MRI, QA, kidney imaging, abdominal imaging, physiology, hydration

## Abstract

**Introduction:** Magnetic resonance elastography (MRE) is a non-invasive method to quantify biomechanical properties of human tissues. It has potential in diagnosis and monitoring of kidney disease, if established in clinical practice. The interplay of flow and volume changes in renal vessels, tubule, urinary collection system and interstitium is complex, but physiological ranges of *in vivo* viscoelastic properties during fasting and hydration have never been investigated in all gross anatomical segments simultaneously.

**Method:** Ten healthy volunteers underwent two imaging sessions, one following a 12-hour fasting period and the second after a drinking challenge of >10 mL per kg body weight (60–75 min before the second examination). High-resolution renal MRE was performed using a novel driver with rotating eccentric mass placed at the posterior-lateral wall to couple waves (50 Hz) to the kidney. The biomechanical parameters, shear wave speed (c_s_ in m/s), storage modulus (G_d_ in kPa), loss modulus (G_l_ in kPa), phase angle $\left(\Upsilon =\frac{2}{\pi }\mathrm{atan}\frac{G_{l}}{G_{d}}\right)$ and attenuation (α in 1/mm) were derived. Accurate separation of gross anatomical segments was applied in post-processing (whole kidney, cortex, medulla, sinus, vessel).

**Results:** High-quality shear waves coupled into all gross anatomical segments of the kidney (mean shear wave displacement: 163 ± 47 μm, mean contamination of second upper harmonics <23%, curl/divergence: 4.3 ± 0.8). Regardless of the hydration state, median G_d_ of the cortex and medulla (0.68 ± 0.11 kPa) was significantly higher than that of the sinus and vessels (0.48 ± 0.06 kPa), and consistently, significant differences were found in c_s_, Υ , and G_l_ (all *p* < 0.001). The viscoelastic parameters of cortex and medulla were not significantly different. After hydration sinus exhibited a small but significant reduction in median G_d_ by −0.02 ± 0.04 kPa (*p* = 0.01), and, consequently, the cortico-sinusoidal-difference in G_d_ increased by 0.04 ± 0.07 kPa (*p* = 0.05). Only upon hydration, the attenuation in vessels became lower (0.084 ± 0.013 1/mm) and differed significantly from the whole kidney (0.095 ± 0.007 1/mm, *p* = 0.01).

**Conclusion:** High-resolution renal MRE with an innovative driver and well-defined 3D segmentation can resolve all renal segments, especially when including the sinus in the analysis. Even after a prolonged hydration period the approach is sensitive to small hydration-related changes in the sinus and in the cortico-sinusoidal-difference.

## Introduction

Acute and chronic kidney diseases are a global burden to healthcare systems worldwide due to their disproportionate ratio between relative low incidence and high care costs (Bello et al., 2017). While early diagnosis and treatment are key to tackling this challenge in the short term (Stack et al., 2014; Bello et al., 2017), urgently needed improvements in therapeutic options are expected to follow from new pathophysiological insights (Fine and Norman, 2008). Medical imaging has already shown its potential to improve diagnosis over the last decades (Selby et al., 2018; Bane et al., 2023; Päivärinta et al., 2023).

### Kidney function

Kidneys participate in many vital physiological processes. In brief, the formation of urine to maintain fluid balance, acid-base and electrolytes homeostasis, clearance of toxins, as well as blood pressure, and hormone synthesis and excretion (regulation). This is also reflected by extraordinary physiological properties. For example, both kidneys consume around 20% of the cardiac output, which leads to a cortical blood perfusion of around 400–500 mL/min/100 g, and medullary perfusion of around 100–150 mL/min/100 g. Enclosed in rigid renal capsules, the kidneys are literally under pressure. This hydrostatic pressure enables a relatively constant filtration of blood in the glomeruli, i.e., glomerular filtration rate (∼120 mL/min; GFR). Therefore, quantifying biomechanical properties—even with contributions from perfusion and fibrosis—has the potential to assess kidney function (Marticorena Garcia et al., 2016; Kirpalani et al., 2017; Marticorena Garcia et al., 2018a; Lang et al., 2019; Beck-Tölly et al., 2020; Brown et al., 2020; Zhang et al., 2021; Chen et al., 2022; Shatil et al., 2022).

### Magnetic resonance elastography

Biomechanical properties have been used to differentiate between healthy and diseased tissues since the beginning of the art of healing, and can be found in historical scientific literature, e.g., in Auenbrugger’s ‘Inventum Novum’ describing percussion from 1754 (Bishop, 1961). Methods for non-invasive quantitative imaging of elastic tissue properties, i.e., elastography, were developed based on ultrasound (Ophir et al., 1991), and magnetic resonance imaging (Muthupillai et al., 1995; Manduca et al., 2021). Ultrasound-based elastography is a fast clinical tool with high spatial resolution (Marticorena Garcia et al., 2018a) that is especially suitable for imaging renal transplants situated superficially in the iliac fossa (Correas et al., 2016). However, tissue anisotropy may influence ultrasound-based estimates of biomechanical properties (Gennisson et al., 2012), and its application is limited by the low penetration depth (Kennedy et al., 2020), transducer application pressure (Syversveen et al., 2012; Kennedy et al., 2020), and intra- and interobserver variability. In contrast, magnetic resonance elastography (MRE) mitigates some of these limitations, as was demonstrated in several studies comparing the two methods in patients with renal transplants (Marticorena Garcia et al., 2018a; Kennedy et al., 2020; Elsingergy et al., 2023), with some variability regarding their diagnostic value. When performing MRE, typically, a stationary acoustic wave source is attached to the human body, and a synchronized phase-sensitive magnetic resonance imaging (MRI) sequence is used to capture the shear wave propagating through the tissue of interest, exploiting the phase accrual caused by the displacement of tissue during the application of motion encoding gradients (MEG). The viscoelastic properties of the tissue can then be estimated from the displacement field by inverting the three-dimensional wave equation, which usually requires smoothing or regularization and often employs removal of the divergence term to decouple the shear wave from compressional contributions (Hirsch et al., 2017; Manduca et al., 2021).

In the last decades the organ studied most frequently by MRE was the liver (Sinkus et al., 2018; Allen et al., 2020; Ehman, 2022; Darwish et al., 2023), although, interestingly the kidneys were already mentioned in the seminal article (Muthupillai et al., 1995). Since then, MRE methods have been improved and have shed light on the potential to assess (patho-) physiological renal processes (Morrell et al., 2017). According to a Pubmed search, 20 October 2023, we could identify 26 studies on the human kidney (excluding animal, *ex-vivo* and tumor studies) (Bensamoun et al., 2011; Rouvière et al., 2011; Lee et al., 2012; Streitberger et al., 2014; Low et al., 2015; Marticorena Garcia et al., 2016; Dittmann et al., 2017; Kirpalani et al., 2017; Marticorena Garcia et al., 2018a; Marticorena Garcia et al., 2018b; Kline et al., 2018; Gandhi et al., 2019; Lang et al., 2019; Marticorena Garcia et al., 2019; Brown et al., 2020; Gandhi et al., 2020; Han et al., 2020; Kennedy et al., 2020; Marticorena Garcia et al., 2021; Zhang et al., 2021; Dillman et al., 2022; Güven et al., 2022; Shatil et al., 2022; Chen et al., 2023a; Chen et al., 2023b; Elsingergy et al., 2023). So far, only pneumatic (Bensamoun et al., 2011; Rouvière et al., 2011; Lee et al., 2012; Low et al., 2015; Marticorena Garcia et al., 2016; Dittmann et al., 2017; Kirpalani et al., 2017; Marticorena Garcia et al., 2018a; Marticorena Garcia et al., 2018b; Kline et al., 2018; Gandhi et al., 2019; Lang et al., 2019; Marticorena Garcia et al., 2019; Brown et al., 2020; Gandhi et al., 2020; Han et al., 2020; Kennedy et al., 2020; Marticorena Garcia et al., 2021; Zhang et al., 2021; Dillman et al., 2022; Güven et al., 2022; Shatil et al., 2022; Chen et al., 2023a; Chen et al., 2023b; Elsingergy et al., 2023), or piezoelectric drivers (Streitberger et al., 2014) were used. From those, 18 studies included healthy subjects, i.e., native kidneys (Bensamoun et al., 2011; Rouvière et al., 2011; Streitberger et al., 2014; Low et al., 2015; Marticorena Garcia et al., 2016; Dittmann et al., 2017; Marticorena Garcia et al., 2018b; Kline et al., 2018; Gandhi et al., 2019; Lang et al., 2019; Marticorena Garcia et al., 2019; Brown et al., 2020; Gandhi et al., 2020; Han et al., 2020; Dillman et al., 2022; Chen et al., 2023a; Chen et al., 2023b; Elsingergy et al., 2023) including on median 12 healthy participants, and a mean voxel volume of 59 ± 36 mm³; breath-hold acquisitions were applied in only eight studies (Bensamoun et al., 2011; Rouvière et al., 2011; Low et al., 2015; Marticorena Garcia et al., 2016; Gandhi et al., 2019; Gandhi et al., 2020; Chen et al., 2023a; Chen et al., 2023b), and only three studies applied a fasting and hydration protocol (Dittmann et al., 2017; Marticorena Garcia et al., 2018b; Chen et al., 2023a) and no study applied high-resolution segmentation of all gross anatomical sections of the kidney (including the sinus).

The aim of this exploratory study is to present a high-resolution breath-hold MRE method, employing a novel transducer on native kidneys. Biomechanical properties were estimated specifically for all gross anatomical structures (whole kidney, cortex, medulla, sinus, vessels) after an extended fasting period, and after the application of a standardized hydration protocol. Currently, sinusoidal biomechanics were never estimated under controlled hydration, yet, hydration levels have been reported to modulate stiffness also in other organs (e.g., liver) as described in Ipek-Ugay et al. (2016) and Dittmann et al. (2017). Therefore, hydration levels should be taken into account when exploring potential biomarkers to assess (patho-) physiological processes, especially in the kidney (Selby et al., 2018; Wilson et al., 2020).

## Materials and methods

### Healthy subjects and preparation steps

Ten young, healthy volunteers (5 male and 5 female; mean ± standard deviation, age: 26 ± 5 years, body mass index: 22 ± 2 kg/m^2^, weight: 66 ± 9 kg, systolic blood pressure: 119 ± 10 mmHg, diastolic blood pressure: 76 ± 8 mmHg, heart rate: 65 ± 15 beats per minute; detailed inclusion and exclusion criteria summarized in the Supplementary Materials) were asked to refrain from food and water for 12 h overnight. In the morning, all subjects were asked regarding their fasting period and medical history, with a special focus on the kidney, and their seated blood pressure was measured. Then they were asked to empty their bladder prior to multiparametric MR measurements (total 50 ± 6 min) and MRE measurements. Subjects were then asked to drink water or diluted fruit juice (>10 mL per kg body weight) and to empty their bladder again before all multiparametric MR and MRE measurements were repeated. The mean duration between the end of the first MR measurement and the beginning of the second MR measurement was 20 ± 4 min (where the time included escorting the subjects out of the scanner, the hydration time and toilet break, and repositioning in the scanner). The time between the hydration and the first “hydrated” MRE measurement (right side) took 61 ± 6 min, and the left side was measured 16 ± 6 min later. All subjects were interviewed after each MRE acquisition regarding discomfort or breathing issues.

All subjects gave written, informed consent to their participation prior to their attendance. The study was conducted in accordance with the current version of the Helsinki declaration and was approved by the ethics commission of the Medical University of Vienna (1595/2015).

### MR setup and sequences

**FIGURE 1:**
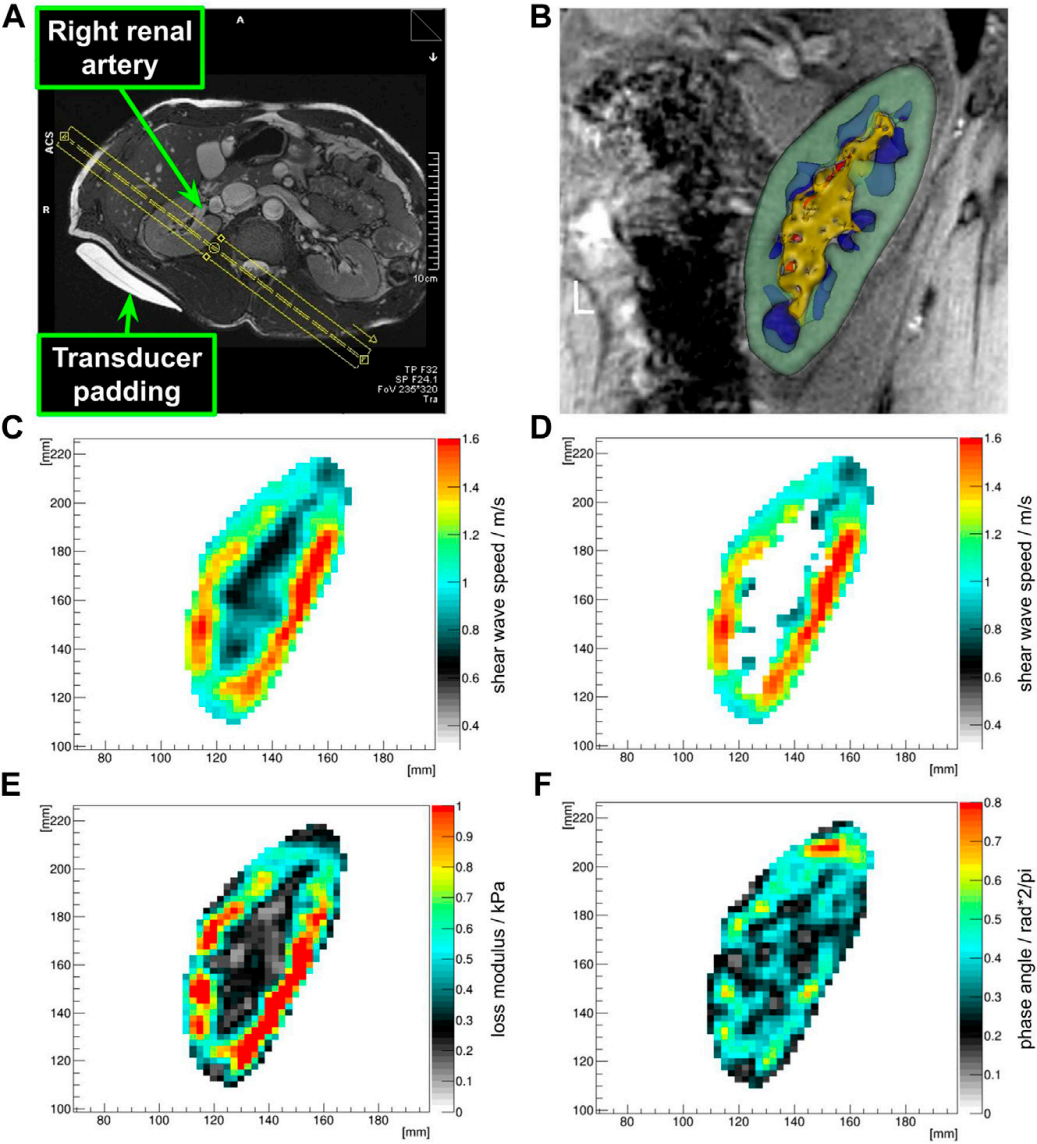
Placement of the MRE field of view **(A)**. The FOV is sagittally rotated to be parallel to the transducer (MR-invisible) and the transducer padding (MR-visible) as well as orthogonal to the renal artery. The center of the FOV in head and foot direction was placed in the middle of the renal sinus. Both renal rims were covered with no folding artifacts. Optimal coverage of the renal poles was ensured. Clipped 3D model after segmentation showing the renal cortex (green), medulla (blue), sinus (yellow), and vessels (red) **(B)**. Shear wave speed map in the kidney ROI **(C)**, and associated renal cortex mask **(D)**, loss modulus map **(E)** and phase angle map **(F)** of the kidney ROI clearly show differences between sinusoidal structures and the cortex and medulla.

All MR measurements were performed on a 3 T whole-body MR system (MAGNETOM Prisma^fit^, Siemens Healthineers, Erlangen, Germany). Two flexible 18-element array coils (“Body 18,” Siemens Healthineers) were placed around the subject’s torso, i.e., in the back and the front. Initially, imaging localizers were used to identify the position of the kidney with respect to the coils. A gravitational transducer [GT, King’s College London, London, United Kingdom; liver transducer, see (Runge et al., 2018)] with a curved contact plate (15 × 11.5 cm) holding a gel pad towards the subjects was placed on the posterior-lateral abdominal wall next to the kidney. On top, a hook-and-loop fastener belt (width = 15 cm) was used to hold the GT in place. First the GT was placed on the right side (Figure 1A), and then—after imaging the right side—on the left side. The GT was driven by a stepper motor and was controlled and synchronized with the MRE sequence via scanner-derived trigger pulses. The rotational motion of the stepper motor located outside the scanner room was transferred via a flexible plastic shaft passing through the scanner room’s waveguide.

A 2D phase contrast gradient-echo (GRE) MRE sequence was applied in four consecutive breath-holds (4 × 20 s, end-expiration), each acquisition with motion encoding gradients (MEG) in one of the three orthogonal directions and one without MEG (Darwish et al., 2023). Four wave phases were captured per direction. The oscillating MEG were applied fractionally to capture two opposing acoustic wave phases (i.e., two k-space lines) per wave period (Rump et al., 2007). MRE acquisition parameters were: 8 slices, voxel size: 2.5 × 2.5 × 2.5 mm³ isotropic, matrix size: 128 × 108, GRAPPA factor 2, bandwidth: 660 Hz/pixel, flip angle: 20°, TR: 85 ms, TE: 7.38 ms, acoustic wave frequency: 50 Hz, MEG strength: 35 mT/m, MEG duration: 6.1 ms. Additional GRE images with higher in-plane resolution, aligned to the MRE field of view (FOV), were acquired for segmenting the kidney (8 slices, voxel size: 1.25 × 1.25 × 2.5 mm³, TR: 75 ms, TE: 4.92 ms).

An optimal field of view was achieved when the anterio-lateral and postero-medial renal rims were equally covered and no relevant folding artifacts occurred. The center of the FOV was positioned on the center of the renal sinus. The sagittal FOV was oriented to be orthogonal to the renal artery. Furthermore, the GT and FOV were placed parallel to each other, so that compressional waves would travel through-plane (Figure 1A).

The in-line reconstruction implemented at the scanner console was used to ensure that data quality was sufficient for post processing. This included shear wave propagation in all three planes (as seen after the application of the curl operator), assessment of non-linearity [defined as percent contamination of destructive second upper harmonics contamination (Runge et al., 2018)], total amplitude of the shear wave displacement in µm, and the ratio of curl over divergence of the displacement field (Manduca et al., 2021).

The MR system vendor’s adaptive-combine coil combination method was used, DICOM magnitude and phase images were exported from the scanner and converted to NIFTI (MRIcron, [Rorden and Brett, 2000)] for post processing in KIR [provided by RS, (Sinkus et al., 2005)]. In short, MRE postprocessing included phase unwrapping, application of a 3D Gaussian filter (σ = 0.75 voxel, kernel size 3 × 3 × 3 voxel), and voxel-wise calculation of viscoelastic tissue properties through direct inversion of the 3D displacement field, after applying the curl operator for removal of compressional components, similar to the method described in (Darwish et al., 2023). Shear wave speed (c_s_ in m/s), storage modulus (G_d_ in kPa), loss modulus (G_l_ in kPa), attenuation (α in 1/mm), and phase angle [ $\Upsilon =\frac{2}{\pi }\mathrm{atan}\frac{G_{l}}{G_{d}}$ ; ranging from purely elastic materials (0) to purely viscous materials (1)] were derived for the two innermost slices.

Renal segmentation was performed manually on the aligned high resolution anatomical images in 3D Slicer (Kikinis et al., 2014); with the following regions of interest (ROIs, see also Figures 1B–D): kidney, cortex, medulla, sinus (includes: renal sinus fat, urinary collection system, vessel with slow flow) and vessel (includes arterial and venous vessels due to their flow-associated hyperintense signals). This was done by MW (>13 years of experience on renal segmentation) and under supervision of GH (>25 years of experience in the field of urogenital radiology). The number of voxels (=volume) was extracted for quality assurance. These ROIs were then used as masks to extract the associated biomechanical properties from the calculated maps. Furthermore, the difference between the ROIs were assessed (cortex—medulla, cortex—sinus, medulla—sinus, cortex—(vessel and sinus), medulla—(vessel and sinus), cortex—vessel, medulla—vessel, sinus—vessel). Associated mean, median, standard deviation and root mean square were summarized for statistical calculations.

**FIGURE 2:**
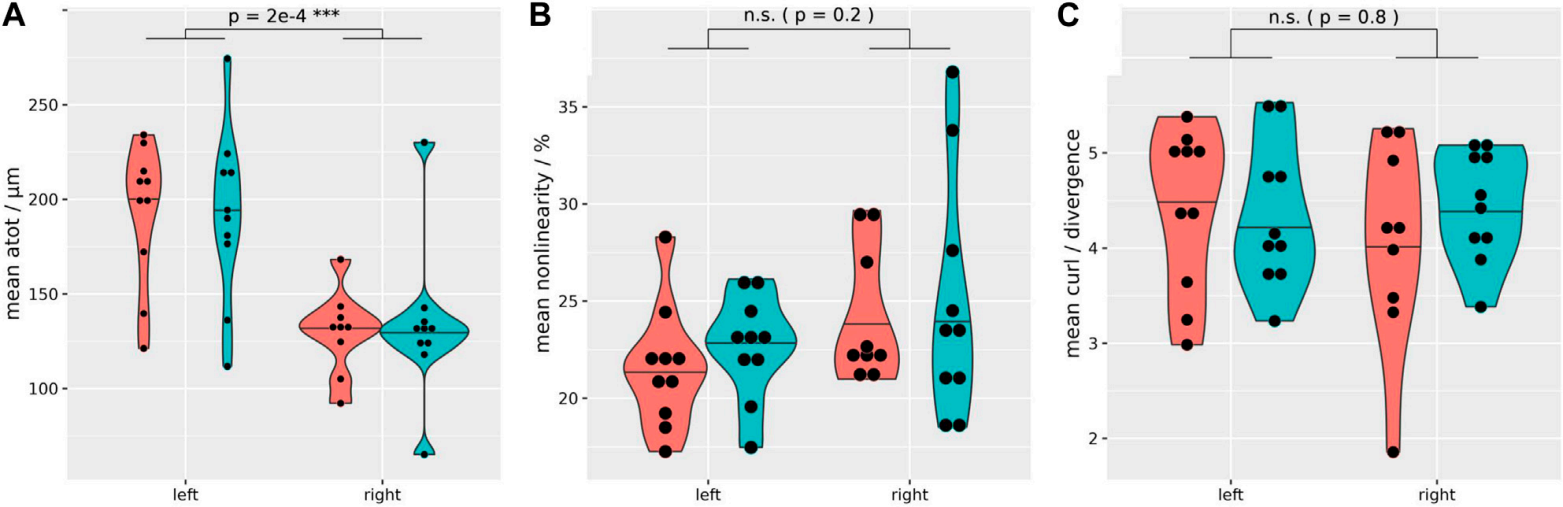
Quality assurance MRE data distribution. Regardless of the hydration state, both kidneys exhibit sufficient wave penetration and quality for biomechanical data calculation (coral = fasting, turquoise = hydrated). However, a significantly higher total shear wave displacement is evident in the left kidney (mean shear wave displacement left: 192 μm, right: 132 µm) **(A)**. Non-linearity **(B)**, and the ratio of curl over divergence **(C)** show no significant difference between the left and right kidney. Paired *t*-test, two-sided, non-equal variances; case 8 excluded. ***p ≤ 0.001.

Paired samples t-tests were applied between the mean sample data of the quality assurance (see Figure 2, ratio curl over divergence, total shear wave displacement, contamination of second upper harmonics in % [as non-linearity]), before and after hydration as well as between the left and right side, to determine performance differences, using R (R Core Team, 2023). Due to the skewed sample data distribution, Wilcoxon rank-sum tests were applied on the median biomechanical sample data to determine significant results. Samples with ties were excluded. The R-package ggplot2 was used for visualization of the results (Wickham, 2009). A *p*-value of less or equal to 0.05 was considered to be significant.

## Results

All subjects managed to comply with the protocol, including the fasting period and the hydration challenge. Initial localization of the kidney allowed for successful placement of the GT closest to the kidney at the posterior-lateral abdominal wall (between the regio vertebralis and lumbalis) of all healthy volunteers. The mean distance between the left and right kidney to the GT pad was 20 and 21 mm, respectively. In contrast, the mean distance from the left and right kidney to the back was 42 and 45 mm, respectively. The MRE images showed no significant misalignment over the consecutive exhaled breath-holds during the MRE acquisition and the high-resolution anatomical scan, so that segmented renal tissues were seamlessly overlaid on biomechanical images (see examples on Figures 1B–F). Also segmented volumes remained relatively stable throughout the study (Supplementary Table S1). Subjects reported no discomfort during the MRE measurements and the prescribed breath-holds were well tolerated. On one subject the trigger box (converting the optical signal from the scanner to the stepper motor via BNC) malfunctioned, so that during the dehydration period the right kidney could not be measured (case 8; external power supply did not work properly). Quality assurance of the MRE data shows a sufficient shear wave penetration and quality in all kidney segments and on both sides, as summarized in Table 1 and Figure 2. However, a significantly higher shear wave displacement was found on the left kidney with *p* = 0.0002 (Figure 2A). Supplementary Figure S1 shows an example data set with phase images and shear wave images (animated files are also shared in the Supplementary Material). At 2.5 × 2.5 × 2.5 mm³ resolution, 50 Hz excitation frequency and a median shear wave speed of 0.96 m/s, this resulted in 7.7 voxels per wavelength.

**TABLE 1:** Quantification of quality assurance MRE data. This table includes all the acquired MRE data, including measurements before and after the drinking challenge (fasting and hydration) as well as both sides (left and right kidney). “Kidneys” subsumes all gross anatomical structures. “VesselSinus” summarizes the ROI of the renal sinus and renal vessels.

Quality assurance								
ROI	Number of voxels		Nonlinearity/%		Total displacement/µm		Curl/divergence	
	Mean	SD	Mean	SD	Mean	SD	Mean	SD
Kidneys	1262	168	23	4	163	47	4.3	0.8
Cortex	662	203	21	4	163	47	4.8	1.0
Medulla	280	70	23	5	165	49	4.1	1.0
Sinus	159	70	32	7	159	48	3.3	0.8
Vessel	60	29	32	7	160	46	3.3	0.9
VesselSinus	218	73	32	7	160	47	3.3	0.7

**FIGURE 3:**
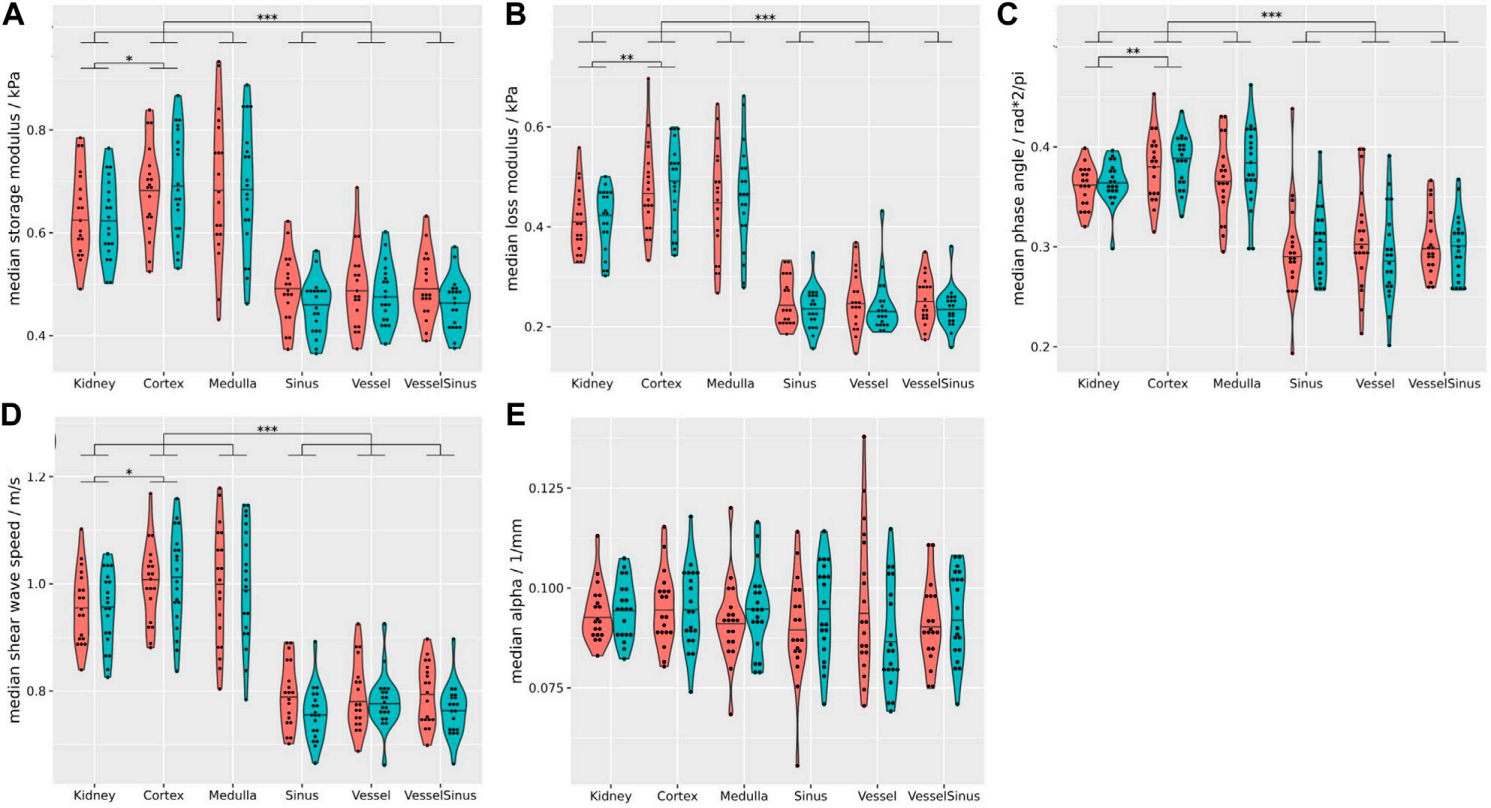
Summary of all biomechanical properties of all renal segments (coral = fasting, turquoise = hydrated). Regardless of the hydration state storage modulus **(A)**, loss modulus **(B)**, phase angle **(C)**, and shear wave speed **(D)** of the sinusoidal structures differ significantly from the cortex, medulla and the whole kidney (*p* ≤ 0.001). Attenuation **(E)** was not significantly different between the gross anatomical segments. Stars over brackets indicate significant differences between pairs of gross anatomical segments. Detailed results and statistical analysis are given in Supplementary Table S6. Wilcoxon rank-sum tests, two-sided, non-paired, and case 8 excluded. *p ≤ 0.05, **p ≤ 0.01, ***p ≤ 0.001.

Figure 3 summarizes the biomechanical properties of all renal segments (data including both hydration states). Regardless of the hydration state, cortex and medulla showed significantly higher median c_s_ (1.01 ± 0.1 m/s), G_d_ (0.68 ± 0.11 kPa), G_l_ (0.47 ± 0.1 kPa) and Υ (0.38 ± 0.04) than the sinusoidal structures (c_s_ = 0.77 ± 0.06 m/s, G_d_ = 0.48 ± 0.06 kPa, G_l_ = 0.24 ± 0.05 kPa, Υ = 0.3 ± 0.05; *p* ≤ 0.001). The cortex exhibited the highest median c_s_ (1.01 ± 0.08 m/s), G_d_ (0.68 ± 0.09 kPa), G_l_ (0.48 ± 0.09 kPa), and Υ (0.39 ± 0.03), while no significant viscoelastic difference was found between cortex and medulla. The attenuation did not differ significantly between the renal segments, but vessels exhibited the lowest attenuation (0.086 ± 0.016 1/mm, Figure 3E). Differences between all segments (before and after hydration as well as summarizing both hydration states), and their statistical analysis are summarized in Supplementary Table S6.

**FIGURE 4:**
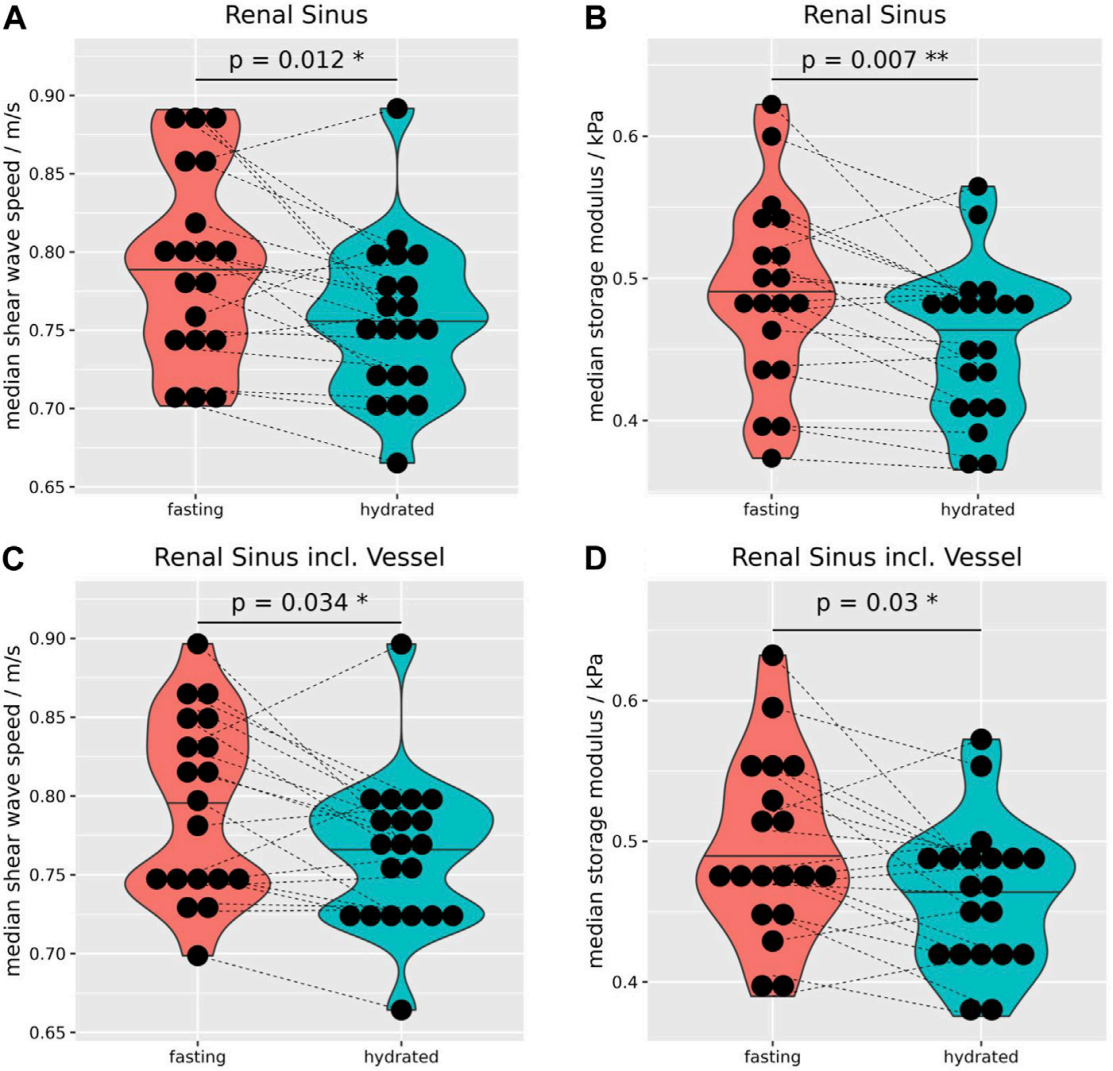
Due to the hydration, median shear wave speed **(A)** and storage modulus **(B)** decreased significantly by −0.03 ± 0.05 m/s, and −0.02 ± 0.04 kPa, respectively. Even when including vessels to the sinus MRE data showed a significant reduction of wave speed by −0.03 ± 0.05 m/s **(C)** and storage modulus by −0.03 ± 0.05 kPa **(D)**. Wilcoxon rank-sum tests, two-sided, paired, and case 8 excluded. *p ≤ 0.05, **p ≤ 0.01.

After hydration the sinus showed a small but significant reduction in c_s_ (−0.03 ± 0.05 m/s, *p* = 0.02), and G_d_ (−0.02 ± 0.04 kPa, *p* = 0.01 Figures 4A, B), and this finding remained significant even when including vessel data, which *per se* exhibited no significant difference (Figures 4C, D). Supplementary Table S2 summarizes the biomechanical properties of the kidneys before and after hydration. The biomechanical properties of the left and right side, before as well as after hydration, are summarized in Supplementary Table S4.

**FIGURE 5:**
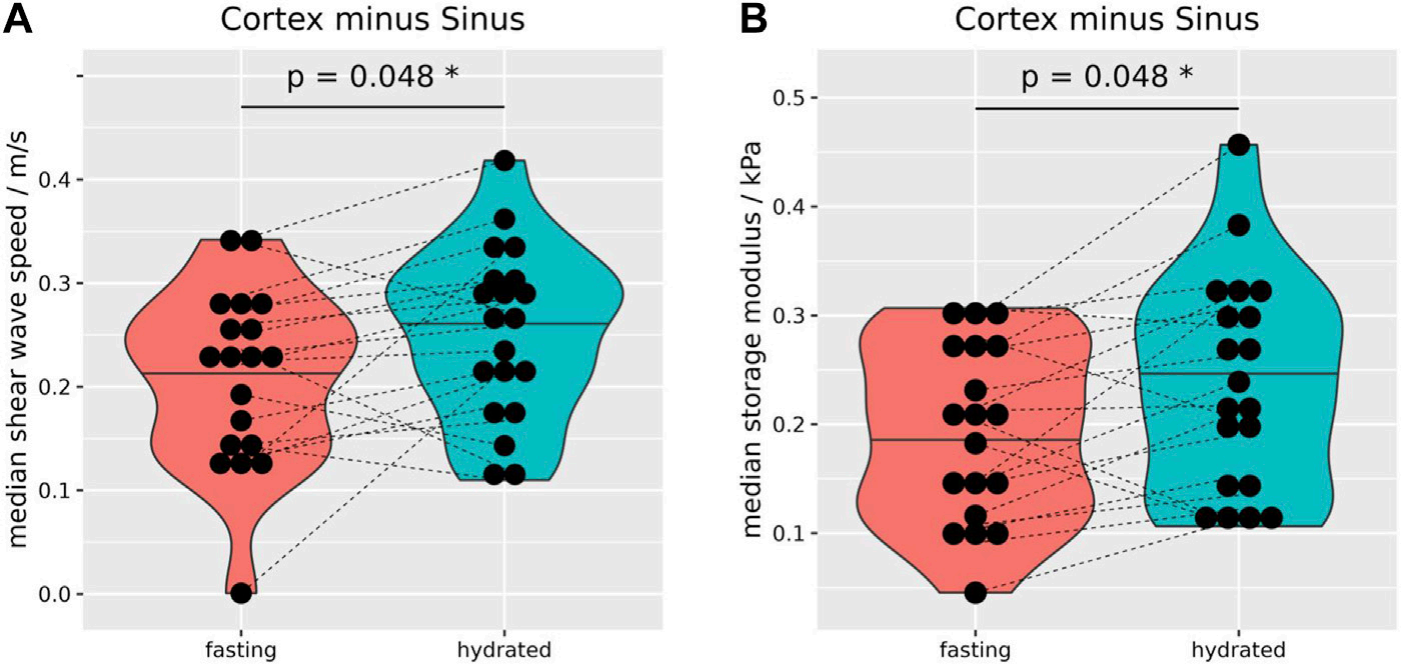
Median differences of shear wave speed **(A)** and storage modulus **(B)** between the renal cortex and sinus. After hydration, differences were significantly increased for shear wave speed (0.04 ± 0.08 m/s, **(A)** and storage modulus (0.04 ± 0.07 kPa, **(B)**. Wilcoxon rank-sum tests, two-sided, paired, and case 8 excluded. *p ≤ 0.05.

As the cortical biomechanical properties remained relatively stable after hydration, the difference of c_s_, and G_d_ between the cortex and sinus increased significantly after hydration (Figures 5A, B). The calculation of biomechanical differences before and after hydration between intrarenal segments of both kidneys are summarized in Supplementary Table S3, and separated by side in Supplementary Table S5.

**FIGURE 6:**
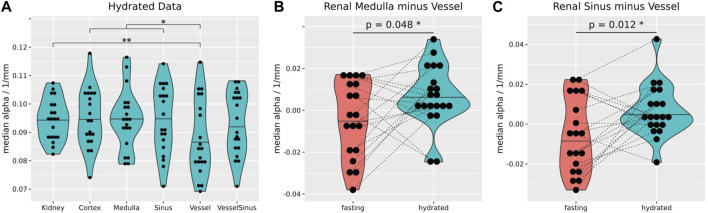
After hydration, the median attenuation in the vessel segment (0.084 ± 0.013 1/mm) was significantly lower than that over the whole kidney (0.095 ± 0.007 1/mm, *p* = 0.01), cortex (0.094 ± 0.01 1/mm, *p* = 0.013) and sinus (0.091 ± 0.012 1/mm, *p* = 0.03) **(A)**. The median difference of attenuation between medulla and vessels **(B)**, and sinus and vessels **(C)** increased significantly upon hydration (0.01 ± 0.02 1/mm and 0.02 ± 0.02 1/mm). Stars over brackets indicate significant differences between pairs of gross anatomical segments. Wilcoxon rank-sum tests, two-sided, non-paired, and case 8 excluded **(A)**. Wilcoxon rank-sum tests, two-sided, paired, and case 8 excluded **(B,C)**. *p ≤ 0.05, **p ≤ 0.01.

After hydration the attenuation of the vessels was significantly reduced in comparison to the whole kidney, cortex, and sinus (Figure 6A). Also, between fasting and hydration the medullary and vessel difference, as well as sinus and vessel difference showed a small but significant increase (Figures 6B, C).

## Discussion

Our method showed a sufficient shear wave quality and penetration into native kidneys to derive various biomechanical properties (Table 1; Figure 2) in all gross anatomical segments (Figure 3). This was achieved by 1) employing a state-of-the-art 3 T scanner to acquire high-resolution renal MRE data, 2) using an innovative driver with a rotating eccentric mass, GT (Runge et al., 2018), 3) choosing a frequency that was sufficiently low to achieve the required penetration depth and high enough for the viscoelastic model to be applicable (Sinkus et al., 2005), and 4) complete segmentation of the kidney through overlaid high-resolution anatomical scans. We consider each of these contributions (using the same numbering) below.

i) High resolution MRE within a feasible breath-hold time was performed with fractional motion encoding (Rump et al., 2007). From the existing literature, no study achieved a smaller voxel volume, and only one method/group, using multifrequency MRE (tomoelastography with weighted shear wave calculations), published seven papers using the same resolution with a voxel volume of 16 mm³ (Streitberger et al., 2014; Marticorena Garcia et al., 2016; Marticorena Garcia et al., 2018a; Marticorena Garcia et al., 2018b; Lang et al., 2019; Marticorena Garcia et al., 2019; Marticorena Garcia et al., 2021), of which only one study deployed breath-hold acquisition similar to that used in this study (Marticorena Garcia et al., 2016). Our rationale to acquire high-resolution data, even with the drawback of reduced SNR, is that the structures within the kidney are relatively small. For example, the mean cortical thickness is reported to be 6 mm (Glodny et al., 2009).
ii) To our knowledge, this is the first study to use a rotating eccentric mass to induce acoustic waves into the kidney (Runge et al., 2018; Manduca et al., 2021). So far, only pneumatic (Bensamoun et al., 2011; Rouvière et al., 2011; Lee et al., 2012; Low et al., 2015; Marticorena Garcia et al., 2016; Dittmann et al., 2017; Kirpalani et al., 2017; Marticorena Garcia et al., 2018a; Marticorena Garcia et al., 2018b; Kline et al., 2018; Gandhi et al., 2019; Lang et al., 2019; Marticorena Garcia et al., 2019; Brown et al., 2020; Gandhi et al., 2020; Han et al., 2020; Kennedy et al., 2020; Marticorena Garcia et al., 2021; Zhang et al., 2021; Dillman et al., 2022; Güven et al., 2022; Shatil et al., 2022; Chen et al., 2023a; Chen et al., 2023b; Elsingergy et al., 2023), or piezoelectric drivers (Streitberger et al., 2014) were used. Only one study reported the total shear displacement from 8–35 μm at 45 and 76 Hz (pneumatic system) (Rouvière et al., 2011). In stark contrast, we achieved a mean shear wave displacement of 163 μm, placing the GT on the posterior-lateral wall, which is the closest to the kidneys. If, in contrast, the subjects would lie directly on the transducer (which was tested in preliminary experiments) adds a few more centimeters between kidney and transducer, resulting in an additional attenuation of the shear wave. Additionally, this would decrease patient comfort, and we observed insufficient coupling of the wave into the subject’s body, possibly due to subconscious arching of the back or rolling to the contralateral side. In our study, shear wave displacement was significantly higher in the left kidney (Figure 2A), which also has not been reported previously. This could be caused by its smaller distance to the GT, and that the contralateral kidney is located close to the liver, which can induce an additional dampening. This finding might suggest the need to reduce the MEG strength on the left side, because high-amplitude shear waves can disturb the phase information and challenge unwrapping, and render the need for using a more complex disturbed-parameter system away from the pure elastic model (Piersol and Paez, 2009; Chen et al., 2023b). To our knowledge, no previous renal MRE study reported the ratio of curl over divergence (quantifying how well the assumption of a pure shear wave field is fulfilled) or non-linearity (indicating the presence of second upper harmonics) (Sinkus et al., 2005; Runge et al., 2018; Manduca et al., 2021). Van Schelt et al. (2023) used a GT to estimate pancreas stiffness and showed comparable results.
iii) The rationale for applying a driver frequency of 50 Hz is manyfold. Firstly, the excitation strength of the GT increases quadratically with the driver frequency, while maintaining a clear frequency spectrum without significant upper harmonics (Table 1; Figure 2) (Runge et al., 2018). Additionally, the cycle duration of 20 ms allowed fractionally encoded MEG to be applied in the sequence, shortening the acquisition to feasible breath-hold durations. Also, 50 Hz resulted in 7.7 voxels per wavelength which was well within the recommended range for MRE (Manduca et al., 2021). Furthermore, lower frequencies are considered to be dominated by a poroelastic model, and frequencies ≥50 Hz are considered to be linked to viscoelastic effects, which we consider to be more relevant for hydration related changes (McGarry et al., 2015). This was also observed in a multifrequency MRE evaluation of abdominal organs, including the kidney (Dittmann et al., 2017). Lastly, using even higher frequencies is prone to higher attenuation, reducing the penetration depth of shear waves; especially on the well protected native kidneys. To our knowledge, besides multi-frequency tomoelastography studies (Streitberger et al., 2014; Marticorena Garcia et al., 2016; Dittmann et al., 2017; Marticorena Garcia et al., 2018b; Lang et al., 2019; Marticorena Garcia et al., 2019; Marticorena Garcia et al., 2021), our study is the only one applying a single frequency of 50 Hz.
iv) Kidneys have a complex inner structure with relatively small volumes (Glodny et al., 2009). Therefore, high-resolution MRE measurements together with high-resolution and contrast-rich anatomical scans were used to segment all gross anatomical segments in the kidneys in three dimensions. Kidney structures were identified based on T_1_ contrast, with a well-defined cortico-medullary differentiation (Wolf et al., 2018; Wolf et al., 2022). The cortex included the cortical rim and renal columns. Vessels (with relatively fast flow) were identified by their bright signal, and where present, were segmented when reaching into the renal columns. The renal sinus had a low signal, which included vessels with slow flow, the urinary collection system, and the renal sinus fat. To our knowledge this is the first study to investigate hydration related biomechanical alterations in all—well segmented—gross anatomical renal segments simultaneously. This is in contrast to many previous studies which used either point-like or simple polygon-shaped ROIs, sometimes defined on single slices only (Rouvière et al., 2011; Dittmann et al., 2017; Brown et al., 2020; Han et al., 2020; Dillman et al., 2022; Güven et al., 2022), or combined different renal structures within segments (Bensamoun et al., 2011; Lee et al., 2012; Streitberger et al., 2014; Low et al., 2015; Marticorena Garcia et al., 2016; Kirpalani et al., 2017; Kline et al., 2018; Gandhi et al., 2019; Lang et al., 2019; Gandhi et al., 2020; Han et al., 2020; Marticorena Garcia et al., 2021; Shatil et al., 2022; Chen et al., 2023b; Elsingergy et al., 2023). Only Marticorena Garcia et al. [in two studies (Marticorena Garcia et al., 2018b; Marticorena Garcia et al., 2019)], and Chen et al. (2023a) used comparably well segmented renal structures. However, Marticorena Garcia et al. used T_2_ weighted images for the segmentation in both studies, although these are known to have poor corticomedullary differentiation (Wolf et al., 2018; Dekkers et al., 2020). And, Chen et al. used the relatively low resolution magnitude MRE images to segment the kidneys. However, it is known that the corticomedullary differentiation is often reduced in renal disease (Wolf et al., 2018; Dekkers et al., 2020), which is a challenge in patient studies (Lee et al., 2012; Marticorena Garcia et al., 2016; Kirpalani et al., 2017; Kline et al., 2018; Lang et al., 2019; Marticorena Garcia et al., 2019; Brown et al., 2020; Han et al., 2020; Marticorena Garcia et al., 2021; Zhang et al., 2021; Dillman et al., 2022; Güven et al., 2022; Elsingergy et al., 2023). Better segmentation could be achieved by employing additional high-resolution T_2_-weighted images, which could distinguish, e.g., the urinary collecting system.

### Biomechanical properties of kidney

We present a subject preparation protocol and MRE method that provides high-resolution and high-quality renal MRE data, which enables us to quantify biomechanical properties in all renal segments. To our knowledge we present the first study in which all renal structures (incl. the sinus) were segmented and evaluated, with a well-controlled fasting and hydration challenge.

The renal sinus has been rarely evaluated in healthy humans using MRE. Arguably, due to the low shear wave quality achieved in some pneumatic MRE studies, indicated by confidence maps marking large areas as non-reliable for quantification, especially in the sinus (Han et al., 2020; Dillman et al., 2022; Shatil et al., 2022; Elsingergy et al., 2023). In contrast, we achieved excellent shear wave quality in all gross anatomical segments in the kidney (Figure 2; Table 1). We found that regardless of the hydration state, sinusoidal c_s_, G_d_, G_i_ and Υ were significantly smaller compared to the cortex and medulla (Figure 3, *p* < 0.001). Only three studies have reported renal sinus *stiffness* measurements, but without applying a concrete fasting or hydration protocol. Contrary to our findings, Bensamoun et al. (2011) found *stiffness* in increasing order from cortex to medulla to sinus. However, they employed very large voxels (100 mm³), and no details were given on segmentation. Consistently with our findings, Streitberger et al. (2014) and Marticorena Garcia et al. (2018b) showed *stiffness* in increasing order from sinus to medulla to cortex. However, we found no biomechanical differences between the cortex and medulla during either fasting or hydration. Regardless of the hydration state, four studies showed that the medulla was *stiffer* than the cortex (Bensamoun et al., 2011; Rouvière et al., 2011; Streitberger et al., 2014; Gandhi et al., 2019), and four studies found that the medulla was *less stiff* than the cortex (Dittmann et al., 2017; Marticorena Garcia et al., 2018b; Gandhi et al., 2020; Dillman et al., 2022). These contradictory findings can be explained by the lack of well-defined segmentations, resolution, and the application of different methods. On the latter, for example, Rouvière et al. showed that a corticomedullary difference was only measurable at 45 Hz but not at 76 Hz (Rouvière et al., 2011). Indeed, the complex shear modulus G* biological tissue is strongly dispersive (Papazoglou et al., 2012), i.e., the frequency of the shear wave use in MRE has a strong influence on the quantified viscoelastic parameters.

### Hydration-related changes

It is known that the need for water intake and the associated water turnover shows a large individual variation depending on age, weight, sex, energy expenditure, physical activity, diet, genetics and environment (Yamada et al., 2022). Therefore, we applied a 12-h overnight fasting period to ensure a well-defined “dehydration” state. To our knowledge, only two studies have applied a prolonged fasting period, “overnight” (Dittmann et al., 2017), and for 10–11-h (Kline et al., 2018), and only three studies employed a drinking challenge at all, either after fasting “overnight” (Dittmann et al., 2017), or for 2 h (Marticorena Garcia et al., 2018b; Chen et al., 2023a) (administering 1 L water). However, Dittmann et al. (2017) and Marticorena Garcia et al. (2018b) presented no detailed timetable regarding the time delay between the hydration challenge and the MRE measurement. Similar to our findings, Dittmann et al. found no hydration-related changes in the cortex or medulla or in the combined corticomedullary dataset (Dittmann et al., 2017), although no well-defined renal segmentation was performed. Marticorena Garcia et al. observed a weak increase in renal column shear wave speed and a weak decrease in medullary shear wave speed after the hydration challenge (and no effect on the cortical rim) (Marticorena Garcia et al., 2018b), however, measurements were performed during free breathing and “moving boundary conditions” between the renal column and medulla could have influenced their data. Chen et al. showed that 30 min after the hydration challenge subjects showed an increase in cortical and medullary *stiffness* and the cortico-medullary ratio changed significantly on both kidneys at 60 Hz (but at 90 Hz only on the right side). However, ROI were drawn on magnitude and wave images of the MRE data set with a relatively large voxel volume: 76 mm³ (Chen et al., 2023a). Also, their time frame was different to ours. Clearly, different biomechanical changes occur at different time points and in different segments. Without any prior food or water restriction, one study investigated the impact of drinking 1 L on healthy subjects, and observed only a significant increase in cortex *stiffness* after 18–22 min (Gandhi et al., 2020). In contrast to all previous studies, our hydration challenge was based on body weight (10 mL/kg of body weight) to further standardize the hydration challenge. This amount is considered feasible for healthy subjects as well as patients with kidney impairment, which allows for comparison in future studies.

We could show that the sinus and vessel data still hold important information regarding the hydration state, even after a time delay of 60–75 min. A possible explanation is that the kidneys sustained their physiological tasks more easily after the hydration, e.g., fluid balance. This could lead to a reduced vascular tonus, decreasing the intramural wall pressure. This is suggested by the small but significant reduction in c_s_ and G_d_ of the sinus (Figures 4A, B). As the cortical biomechanical properties remained relatively stable, the difference in c_s_ and G_d_ between the cortex and sinus increased significantly after hydration (Figures 5A, B). When including big renal vessels (with relatively fast flow; ROI: “VesselSinus”), the c_s_ and G_d_ still decreased significantly upon the hydration challenge in the sinus (Figures 4C, D). This could be linked, on the one hand, to the relatively small volume of the vessels, and on the other hand, to the stable perfusion of kidneys in healthy subjects. Surely, the latter could be more pronounced in patients with hypertension or pre- and post-renal obstructions.

However, sinusoidal biomechanical modulations can be linked to a complex interplay between the small renal arteries and veins (with relatively low flow; vascular tonus, flow and volume changes), the urinary collection system (volume and pressure changes), as our renal sinus segmentation included these structures. Previously, it was reported that sinus *stiffness* increased with a full bladder (Streitberger et al., 2014; Marticorena Garcia et al., 2018b). This was also observed in an ultrasound-based elastography in pigs (Gennisson et al., 2012). Arguably, as our subjects were asked to empty their bladder before each measurement session (before measuring the fasting state, and hydration), and because they did not express a need to empty their bladder during the scanning time, the pressure from the bladder might have not affected the renal sinus.

Regardless of the low standard deviation within the renal segments of our acquired biomechanical data, it would be still favorable to be able to adjust findings to an intrasubject base level. With respect to elasticity, our results support that cortical structures seem to hold promising properties (Figure 5) (Caroli et al., 2018; Pruijm et al., 2018; Selby et al., 2018; Wolf et al., 2018).

During fasting, the attenuation coefficient of all renal segments were not distinguishable (Figure 3E). But after hydration the vessels showed a significant reduction in attenuation (Figure 6A), and the difference between the medulla and vessels (Figure 6B) and sinus and vessel (Figure 6C) exhibited a significant increase within subjects. This could be linked to boundary conditions because of the small volume of the vessels. Furthermore, the findings could be linked to changes in the diameter of the vessels.

Hydration can be considered to alter volumetric and pressure changes in the vessels, in the tubule system, in the urinary collection system as well as in the interstitial compartment; and their (patho) physiological interplay is complex (Fine and Norman, 2008; Niendorf et al., 2015; Selby et al., 2018). For example, even the denervation of renal transplants could induce variations in derived *stiffness* values due to changes in the vascular tonus (Marticorena Garcia et al., 2016). A positive correlation of blood pressure to cortical stiffness was reported by Dillman et al. (2022). This becomes more complicated when fibrosis is present, as it reduces and alters the volume and pressure of the vessels and the tubule system. Therefore, reduced *stiffness* can be linked to reduced blood flow, a surrogate for fibrosis, which *per se* increases *stiffness* (Lee et al., 2012; Kline et al., 2018; Lang et al., 2019; Marticorena Garcia et al., 2019; Brown et al., 2020; Zhang et al., 2021; Güven et al., 2022). But other studies found an increase in *stiffness* with (suggested) higher levels of fibrosis (Marticorena Garcia et al., 2016; Kirpalani et al., 2017; Han et al., 2020; Elsingergy et al., 2023) or inflammation (Shatil et al., 2022). Therefore, measuring fasting and hydration related changes in patients could be a useful tool to unveil insights into pathophysiological processes.

### Limitations

Our study has several limitations. First, we present a small sample size to describe our method and findings. This is mostly due to the demanding fasting and hydration protocol, which made it difficult to recruit participants. Indeed, fasting for such a long period is challenging for patients, especially in the context of renal failure, diabetes mellitus (diabetic kidney disease), patients undergoing dialysis or transplanted patients. Furthermore, the hydration protocol might need to be further standardized for patients, for example, by taking body height and subject age into account. Also the end-expiration breath-hold duration of 20 s is often an insurmountable challenge for (older) patients. We observed that healthy subjects easily tolerate this challenge, which keeps the abdominal organ position more stable throughout multiple breath-holds. For patients with breathing issues, end-inspiration acquisitions might be more appropriate. However, faster MRE methods are currently being investigated (Rump et al., 2007; Guenthner et al., 2019; Darwish et al., 2023). Furthermore, we considered for our MRE models that all renal tissues possess isotropic properties; similar to all previous renal MRE investigations. However, diffusion-weighted images demonstrate that the medulla has relatively strong anisotropic properties (Qin et al., 2013; Caroli et al., 2018; Ljimani et al., 2020). Potential mitigation strategies were envisioned by Sinkus et al. (2000) and Qin et al. (2013). Further investigations have to verify whether this assumption will hold true. Lastly, we did not apply eGFR measurements on our young and healthy group of volunteers, because eGFR is known to be less sensitive in healthy subjects. However, we asked all subjects to give their medical history relevant to the study. In order to detect any relevant renal function differences, our young subjects would have needed to undergo disproportional and invasive methods, such as measured GFR (Selby et al., 2018). Though for patients, Zhang et al. (2021) found that analyzing MRE data together with eGFR could be used to help clinicians in monitoring renal transplants.

## Conclusion

High-resolution renal MRE together with an innovative rotating eccentric mass transducer, and defined 3D segmentation resolved all gross anatomical renal segments. Even after a prolonged period after hydration, our method showed a small but significant reduction in shear wave speed and storage modulus of the sinus. Therefore, well-defined hydration protocols should be considered in future clinical renal MRE investigations to assess the complex (patho) physiological processes in renal disease.

## Data Availability

The original contributions presented in the study are included in the article/Supplementary Material, further inquiries can be directed to the corresponding author.

## Supplementary material

The Supplementary Material for this article can be found online at: https://www.frontiersin.org/articles/10.3389/fphys.2024.1327407/full#supplementary-material

Supplementary Figure S1 Parasagittal T1-weighted magnitude image of the left kidney **(A)** Associated phase image, acquired without MEG **(B)**, acquired with MEG in readout direction **(C)**, in phase direction **(D)**, and in slice direction **(E)**. Shear waves (curl) in readout direction **(G)**, phase direction **(H)** and slice direction **(I)**. Animation of the shear waves **(G–I)** are provided as GIF files in the supplementary materials (Data Sheet 1).
