# Supplementary material for: Magnetic resonance elastography resolving all gross anatomical segments of the kidney during controlled hydration
Source: Front Physiol. 2024 Feb 7;15:1327407. doi: 10.3389/fphys.2024.1327407 (PMC10880033; doi:10.3389/fphys.2024.1327407)
Supplement: Supplementary file 1 [file Table1.DOCX]

## Supplementary Materials

### Inclusion and exclusion criteria

**Inclusion criteria**: 18 to 50 years of age and Body Mass Index (BMI) between 17 and 30 kg/m².

**Exclusion criteria**: mean seated blood pressure above 140/90 mmHg and seated heart rate above 100 beats per second, known atrial fibrillation or atrioventricular block grade II or higher, history of myocardial infarction, unstable angina pectoris, evident congestive heart failure (New York Heart Association class III/IV), diabetes mellitus, established renal impairment or chronic disease and intake of antihypertensive medication, nonsteroidal anti-inflammatory drugs like acetylsalicylic acid or diuretics.

**Summary of Evaluation**

| **ROI measurements** | | | | | | | |
| --- | --- | --- | --- | --- | --- | --- | --- |
| **ROI** | **Hydration State** | **Number of Voxel (Left Kidney)** | | **Number of Voxel (Right Kidney)** | | **Mean relative ROI size change / %** | |
|  |  | mean | SD | mean | SD | mean | p-value |
| **Kidneys** | Dehydration and Hydration | 1334 | 139 | 1178 | 167 | 13% | 0.0035** |
| **Cortex** |  | 711 | 216 | 612 | 185 | 16% | 0.1367 |
| **Medulla** |  | 304 | 67 | 255 | 67 | 19% | 0.0285* |
| **Sinus** |  | 172 | 83 | 144 | 54 | 19% | 0.2415 |
| **Vessel** |  | 59 | 23 | 62 | 36 | -6% | 0.7194 |
| **VesselSinus** |  | 228 | 88 | 206 | 54 | 10% | 0.3777 |
| **Kidneys** | Dehydration | 1314 | 139 | 1169 | 190 | 12% | 0.0718 |
| **Cortex** |  | 718 | 210 | 583 | 206 | 23% | 0.1767 |
| **Medulla** |  | 311 | 48 | 259 | 93 | 20% | 0.1379 |
| **Sinus** |  | 145 | 83 | 131 | 59 | 11% | 0.6845 |
| **Vessel** |  | 61 | 29 | 66 | 36 | -7% | 0.7529 |
| **VesselSinus** |  | 201 | 91 | 197 | 52 | 2% | 0.8988 |
| **Kidneys** | Hydration | 1353 | 144 | 1188 | 152 | 14% | 0.026* |
| **Cortex** |  | 705 | 233 | 640 | 169 | 10% | 0.5005 |
| **Medulla** |  | 298 | 83 | 251 | 29 | 19% | 0.1284 |
| **Sinus** |  | 198 | 77 | 157 | 48 | 26% | 0.1902 |
| **Vessel** |  | 56 | 16 | 58 | 38 | -4% | 0.8657 |
| **VesselSinus** |  | 254 | 81 | 216 | 58 | 18% | 0.2545 |
|  |  | **Mean relative ROI size change in %** | | **Mean relative ROI size change in %** | |  |  |
|  |  | mean | p-value | mean | p-value |  |  |
| **Kidneys** | Dehydration vs Hydration | -3% | 0.5455 | -2% | 0.8210 |  |  |
| **Cortex** |  | 2% | 0.8940 | -9% | 0.5336 |  |  |
| **Medulla** |  | 4% | 0.6908 | 3% | 0.8211 |  |  |
| **Sinus** |  | -27% | 0.1536 | -17% | 0.3106 |  |  |
| **Vessel** |  | 8% | 0.6662 | 12% | 0.6871 |  |  |
| **VesselSinus** |  | -21% | 0.1822 | -9% | 0.4648 |  |  |

**Supplementary Table 1** Summary of ROI volume changes between the left and right kidney and relative changes between the fastening and hydrated state. Non-paired samples t-test used to identify significant results. * p ≤ 0.05, ** p ≤ 0.01.

| **ROI** | **Shear wave speed / m/s** | | **Alpha / 1/mm** | | **Storage modulus / kPa** | | **Loss modulus / kPa** | | **Phase-angle / rad * 2/pi** | |
| --- | --- | --- | --- | --- | --- | --- | --- | --- | --- | --- |
|  | median | SD | median | SD | median | SD | median | SD | median | SD |
| **Both Kidney - Dehydration** | | | | | | | | | | |
| **Kidneys** | 0.95 | 0.07 | 0.092 | 0.007 | 0.62 | 0.08 | 0.40 | 0.06 | 0.362 | 0.020 |
| **Cortex** | 1.01 | 0.07 | 0.093 | 0.009 | 0.69 | 0.09 | 0.45 | 0.09 | 0.384 | 0.034 |
| **Medulla** | 1.01 | 0.11 | 0.092 | 0.010 | 0.68 | 0.14 | 0.45 | 0.11 | 0.364 | 0.039 |
| **Sinus** | 0.79*§ | 0.06 | 0.087 | 0.013 | 0.49*§ | 0.07 | 0.23 | 0.05 | 0.289 | 0.050 |
| **Vessel** | 0.77 | 0.06 | 0.091 | 0.018 | 0.48 | 0.08 | 0.25 | 0.06 | 0.300 | 0.051 |
| **VesselSinus** | 0.80 | 0.06 | 0.089 | 0.010 | 0.48 | 0.06 | 0.24 | 0.05 | 0.293 | 0.032 |
| **Both Kidney - Hydration** | | | | | | | | | | |
| **Kidneys** | 0.96 | 0.07 | 0.095 | 0.007 | 0.62 | 0.08 | 0.43 | 0.06 | 0.363 | 0.022 |
| **Cortex** | 1.02 | 0.09 | 0.094 | 0.010 | 0.68 | 0.10 | 0.50 | 0.08 | 0.390 | 0.026 |
| **Medulla** | 1.00 | 0.11 | 0.094 | 0.010 | 0.68 | 0.12 | 0.46 | 0.10 | 0.379 | 0.041 |
| **Sinus** | 0.76* | 0.05 | 0.094 | 0.012 | 0.46* | 0.05 | 0.24 | 0.04 | 0.302 | 0.038 |
| **Vessel** | 0.77 | 0.05 | 0.084 | 0.013 | 0.47 | 0.06 | 0.23 | 0.06 | 0.288 | 0.046 |
| **VesselSinus** | 0.77§ | 0.05 | 0.092 | 0.011 | 0.47§ | 0.05 | 0.23 | 0.04 | 0.298 | 0.032 |

**Supplementary Table 2** Summarizing the biomechanical properties of both kidneys before and after hydration. * and § show significant changes due to hydration within columns; Wilcoxon rank-sum tests, two-sided, paired, and case 8 excluded.

| **ROI** | **Shear wave speed / m/s** | | **Alpha / 1/mm** | | **Storage modulus / kPa** | | **Loss modulus / kPa** | | **Phase-angle / rad * 2/pi** | |
| --- | --- | --- | --- | --- | --- | --- | --- | --- | --- | --- |
|  | median | SD | median | SD | median | SD | median | SD | median | SD |
| **Both Kidney - Hydration** | | | | | | | | | | |
| **Cortex-Medulla** | 0.03 | 0.08 | -0.001 | 0.011 | -0.02 | 0.10 | 0.03 | 0.10 | 0.023 | 0.050 |
| **Cortex-Sinus** | 0.22* | 0.09 | 0.002 | 0.017 | 0.2* | 0.08 | 0.23 | 0.09 | 0.091 | 0.070 |
| **Medulla-Sinus** | 0.22 | 0.11 | 0.000 | 0.016 | 0.21 | 0.13 | 0.21 | 0.12 | 0.088 | 0.065 |
| **Cortex-VesselSinus** | 0.20 | 0.08 | 0.002 | 0.015 | 0.22 | 0.08 | 0.22 | 0.09 | 0.087 | 0.052 |
| **Medulla-VesselSinus** | 0.22 | 0.10 | 0.000 | 0.012 | 0.21 | 0.12 | 0.20 | 0.11 | 0.077 | 0.047 |
| **Cortex-Vessel** | 0.21 | 0.08 | 0.004 | 0.021 | 0.21 | 0.09 | 0.21 | 0.08 | 0.084 | 0.063 |
| **Medulla-Vessel** | 0.22 | 0.10 | -0.003 | 0.018 | 0.20 | 0.11 | 0.17 | 0.11 | 0.061 | 0.056 |
| **Sinus-Vessel** | 0.01 | 0.05 | -0.005 | 0.019 | 0.00 | 0.05 | -0.02 | 0.05 | -0.006 | 0.056 |
| **Both Kidney - Dehydration** | | | | | | | | | | |
| **Cortex-Medulla** | 0.01 | 0.07 | -0.001 | 0.013 | -0.01 | 0.09 | 0.05 | 0.08 | -0.004 | 0.045 |
| **Cortex-Sinus** | 0.27* | 0.08 | -0.001 | 0.015 | 0.23* | 0.10 | 0.28 | 0.09 | 0.097 | 0.048 |
| **Medulla-Sinus** | 0.22 | 0.10 | 0.001 | 0.016 | 0.22 | 0.11 | 0.22 | 0.10 | 0.099 | 0.064 |
| **Cortex-VesselSinus** | 0.26 | 0.08 | 0.001 | 0.013 | 0.22 | 0.10 | 0.27 | 0.09 | 0.100 | 0.044 |
| **Medulla-VesselSinus** | 0.22 | 0.10 | 0.006 | 0.015 | 0.21 | 0.11 | 0.22 | 0.10 | 0.087 | 0.058 |
| **Cortex-Vessel** | 0.24 | 0.09 | 0.009 | 0.013 | 0.18 | 0.09 | 0.25 | 0.10 | 0.113 | 0.055 |
| **Medulla-Vessel** | 0.22 | 0.10 | 0.005 | 0.015 | 0.20 | 0.09 | 0.24 | 0.10 | 0.108 | 0.061 |
| **Sinus-Vessel** | -0.02 | 0.03 | 0.004 | 0.013 | -0.02 | 0.04 | -0.01 | 0.04 | 0.016 | 0.056 |

**Supplementary Table 3** Summary of biomechanical differences between intra-renal segmentation before and after hydration. * and § show significant changes within the columns; Wilcoxon rank-sum tests, two-sided, paired, and case 8 excluded.

| **Left Kidney - Dehydration** | | | | | | | | | | |
| --- | --- | --- | --- | --- | --- | --- | --- | --- | --- | --- |
| **ROI** | **Shear wave speed / m/s** | | **Alpha / 1/mm** | | **Storage modulus / kPa** | | **Loss modulus / kPa** | | **Phase-angle / rad * 2/pi** | |
|  | median | SD | median | SD | median | SD | median | SD | median | SD |
| **Kidneys** | 1.00 | 0.07 | 0.091 | 0.004 | 0.70 | 0.08 | 0.45 | 0.07 | 0.368 | 0.015 |
| **Cortex** | 1.02 | 0.08 | 0.095 | 0.009 | 0.72 | 0.10 | 0.49 | 0.11 | 0.387 | 0.033 |
| **Medulla** | 1.06 | 0.11 | 0.089 | 0.009 | 0.76 | 0.12 | 0.51 | 0.12 | 0.370 | 0.047 |
| **Sinus** | 0.84 | 0.05 | 0.089 | 0.016 | 0.53 | 0.05 | 0.29 | 0.04 | 0.296 | 0.065 |
| **Vessel** | 0.82 | 0.06 | 0.087 | 0.012 | 0.53 | 0.07 | 0.27 | 0.06 | 0.306 | 0.036 |
| **VesselSinus** | 0.84 | 0.04 | 0.087 | 0.010 | 0.54 | 0.05 | 0.28 | 0.04 | 0.299 | 0.028 |
| **Left Kidney - Hydration** | | | | | | | | | | |
| **ROI** | **Shear wave speed / m/s** | | **Alpha / 1/mm** | | **Storage modulus / kPa** | | **Loss modulus / kPa** | | **Phase-angle / rad * 2/pi** | |
|  | median | SD | median | SD | median | SD | median | SD | median | SD |
| **Kidneys** | 0.99 | 0.07 | 0.095 | 0.008 | 0.66 | 0.09 | 0.45 | 0.06 | 0.372 | 0.017 |
| **Cortex** | 1.04 | 0.09 | 0.096 | 0.011 | 0.73 | 0.12 | 0.50 | 0.09 | 0.399 | 0.022 |
| **Medulla** | 1.05 | 0.13 | 0.093 | 0.011 | 0.75 | 0.14 | 0.49 | 0.12 | 0.369 | 0.047 |
| **Sinus** | 0.78 | 0.05 | 0.097 | 0.012 | 0.49 | 0.04 | 0.26 | 0.04 | 0.305 | 0.036 |
| **Vessel** | 0.80 | 0.05 | 0.085 | 0.011 | 0.50 | 0.05 | 0.25 | 0.07 | 0.294 | 0.041 |
| **VesselSinus** | 0.79 | 0.04 | 0.095 | 0.011 | 0.49 | 0.05 | 0.25 | 0.04 | 0.304 | 0.034 |
| **Right Kidney - Dehydration** | | | | | | | | | | |
| **ROI** | **Shear wave speed / m/s** | | **Alpha / 1/mm** | | **Storage modulus / kPa** | | **Loss modulus / kPa** | | **Phase-angle / rad * 2/pi** | |
|  | median | SD | median | SD | median | SD | median | SD | median | SD |
| **Kidneys** | 0.90 | 0.05 | 0.095 | 0.009 | 0.59 | 0.05 | 0.37 | 0.03 | 0.352 | 0.026 |
| **Cortex** | 0.99 | 0.06 | 0.093 | 0.010 | 0.67 | 0.06 | 0.44 | 0.06 | 0.354 | 0.035 |
| **Medulla** | 0.94 | 0.09 | 0.094 | 0.010 | 0.60 | 0.12 | 0.39 | 0.08 | 0.363 | 0.031 |
| **Sinus** | 0.74 | 0.04 | 0.087 | 0.009 | 0.44 | 0.05 | 0.21 | 0.03 | 0.288 | 0.028 |
| **Vessel** | 0.74 | 0.04 | 0.101 | 0.023 | 0.42 | 0.04 | 0.24 | 0.05 | 0.300 | 0.065 |
| **VesselSinus** | 0.75 | 0.04 | 0.090 | 0.011 | 0.45 | 0.03 | 0.22 | 0.03 | 0.288 | 0.037 |
| **Right Kidney - Hydration** | | | | | | | | | | |
| **ROI** | **Shear wave speed / m/s** | | **Alpha / 1/mm** | | **Storage modulus / kPa** | | **Loss modulus / kPa** | | **Phase-angle / rad * 2/pi** | |
|  | median | SD | median | SD | median | SD | median | SD | median | SD |
| **Kidneys** | 0.95 | 0.07 | 0.095 | 0.007 | 0.60 | 0.06 | 0.41 | 0.06 | 0.360 | 0.024 |
| **Cortex** | 1.00 | 0.09 | 0.094 | 0.009 | 0.67 | 0.09 | 0.50 | 0.09 | 0.368 | 0.025 |
| **Medulla** | 0.99 | 0.09 | 0.095 | 0.010 | 0.67 | 0.11 | 0.46 | 0.10 | 0.389 | 0.037 |
| **Sinus** | 0.72 | 0.04 | 0.094 | 0.012 | 0.42 | 0.04 | 0.22 | 0.03 | 0.297 | 0.041 |
| **Vessel** | 0.76 | 0.04 | 0.080 | 0.016 | 0.44 | 0.05 | 0.21 | 0.02 | 0.269 | 0.050 |
| **VesselSinus** | 0.73 | 0.04 | 0.092 | 0.012 | 0.42 | 0.04 | 0.22 | 0.03 | 0.296 | 0.032 |

**Supplementary Table 4** Summary of biomechanical properties, separated by the left and right kidney as well as by the fastening and hydration state. “VesselSinus” summarizes the ROI of the renal sinus and renal vessels together.

| **Left Kidney - Dehydration** | | | | | | | | | | |
| --- | --- | --- | --- | --- | --- | --- | --- | --- | --- | --- |
| **ROI** | **Shear wave speed / m/s** | | **Alpha / 1/mm** | | **Storage modulus / kPa** | | **Loss modulus / kPa** | | **Phase-angle / rad * 2/pi** | |
|  | median | SD | median | SD | median | SD | median | SD | median | SD |
| **Cortex-Medulla** | -0.018 | 0.077 | 0.010 | 0.014 | -0.056 | 0.075 | -0.003 | 0.118 | 0.029 | 0.063 |
| **Cortex-Sinus** | 0.223 | 0.087 | 0.001 | 0.021 | 0.175 | 0.087 | 0.229 | 0.110 | 0.100 | 0.085 |
| **Medulla-Sinus** | 0.196 | 0.117 | -0.004 | 0.018 | 0.224 | 0.109 | 0.218 | 0.131 | 0.090 | 0.080 |
| **Cortex-VesselSinus** | 0.195 | 0.075 | 0.003 | 0.016 | 0.150 | 0.091 | 0.209 | 0.108 | 0.089 | 0.052 |
| **Medulla-VesselSinus** | 0.213 | 0.104 | -0.005 | 0.013 | 0.229 | 0.110 | 0.211 | 0.124 | 0.064 | 0.052 |
| **Cortex-Vessel** | 0.192 | 0.092 | 0.005 | 0.019 | 0.140 | 0.106 | 0.205 | 0.098 | 0.084 | 0.056 |
| **Medulla-Vessel** | 0.199 | 0.104 | -0.002 | 0.017 | 0.236 | 0.117 | 0.191 | 0.109 | 0.054 | 0.055 |
| **Sinus-Vessel** | 0.002 | 0.061 | -0.005 | 0.016 | 0.007 | 0.037 | 0.002 | 0.049 | -0.006 | 0.053 |
| **Left Kidney - Hydration** | | | | | | | | | | |
| **ROI** | **Shear wave speed / m/s** | | **Alpha / 1/mm** | | **Storage modulus / kPa** | | **Loss modulus / kPa** | | **Phase-angle / rad * 2/pi** | |
|  | median | SD | median | SD | median | SD | median | SD | median | SD |
| **Cortex-Medulla** | 0.011 | 0.066 | 0.005 | 0.015 | -0.031 | 0.083 | 0.041 | 0.069 | 0.011 | 0.053 |
| **Cortex-Sinus** | 0.226 | 0.075 | 0.004 | 0.012 | 0.208 | 0.097 | 0.220 | 0.097 | 0.099 | 0.047 |
| **Medulla-Sinus** | 0.224 | 0.101 | -0.005 | 0.019 | 0.269 | 0.113 | 0.196 | 0.116 | 0.069 | 0.076 |
| **Cortex-VesselSinus** | 0.216 | 0.080 | 0.003 | 0.012 | 0.199 | 0.098 | 0.230 | 0.097 | 0.106 | 0.048 |
| **Medulla-VesselSinus** | 0.222 | 0.107 | -0.003 | 0.018 | 0.268 | 0.112 | 0.189 | 0.114 | 0.071 | 0.071 |
| **Cortex-Vessel** | 0.203 | 0.094 | 0.009 | 0.014 | 0.179 | 0.101 | 0.236 | 0.111 | 0.116 | 0.058 |
| **Medulla-Vessel** | 0.209 | 0.121 | 0.003 | 0.017 | 0.245 | 0.108 | 0.182 | 0.117 | 0.086 | 0.061 |
| **Sinus-Vessel** | -0.024 | 0.036 | 0.004 | 0.011 | -0.021 | 0.021 | -0.011 | 0.049 | 0.020 | 0.040 |
| **Right Kidney - Dehydration** | | | | | | | | | | |
| **ROI** | **Shear wave speed / m/s** | | **Alpha / 1/mm** | | **Storage modulus / kPa** | | **Loss modulus / kPa** | | **Phase-angle / rad * 2/pi** | |
|  | median | SD | median | SD | median | SD | median | SD | median | SD |
| **Cortex-Medulla** | 0.046 | 0.080 | -0.002 | 0.006 | 0.056 | 0.109 | 0.075 | 0.084 | -0.001 | 0.034 |
| **Cortex-Sinus** | 0.250 | 0.081 | 0.002 | 0.013 | 0.215 | 0.074 | 0.230 | 0.080 | 0.079 | 0.054 |
| **Medulla-Sinus** | 0.216 | 0.114 | 0.000 | 0.014 | 0.199 | 0.142 | 0.203 | 0.099 | 0.088 | 0.047 |
| **Cortex-VesselSinus** | 0.245 | 0.074 | -0.001 | 0.014 | 0.217 | 0.070 | 0.219 | 0.073 | 0.061 | 0.055 |
| **Medulla-VesselSinus** | 0.219 | 0.108 | 0.000 | 0.013 | 0.190 | 0.122 | 0.197 | 0.100 | 0.077 | 0.043 |
| **Cortex-Vessel** | 0.242 | 0.070 | -0.003 | 0.024 | 0.217 | 0.081 | 0.216 | 0.064 | 0.088 | 0.073 |
| **Medulla-Vessel** | 0.230 | 0.104 | -0.006 | 0.021 | 0.194 | 0.108 | 0.166 | 0.105 | 0.066 | 0.060 |
| **Sinus-Vessel** | 0.012 | 0.031 | -0.014 | 0.021 | 0.002 | 0.061 | -0.027 | 0.049 | -0.044 | 0.063 |
| **Right Kidney - Hydration** | | | | | | | | | | |
| **ROI** | **Shear wave speed / m/s** | | **Alpha / 1/mm** | | **Storage modulus / kPa** | | **Loss modulus / kPa** | | **Phase-angle / rad * 2/pi** | |
|  | median | SD | median | SD | median | SD | median | SD | median | SD |
| **Cortex-Medulla** | 0.014 | 0.078 | -0.003 | -0.003 | 0.041 | 0.041 | 0.049 | 0.049 | -0.015 | 0.028 |
| **Cortex-Sinus** | 0.292 | 0.090 | -0.004 | -0.004 | 0.269 | 0.269 | 0.303 | 0.303 | 0.081 | 0.050 |
| **Medulla-Sinus** | 0.224 | 0.103 | 0.003 | 0.003 | 0.208 | 0.208 | 0.249 | 0.249 | 0.104 | 0.051 |
| **Cortex-VesselSinus** | 0.280 | 0.086 | -0.004 | -0.004 | 0.256 | 0.256 | 0.295 | 0.295 | 0.087 | 0.040 |
| **Medulla-VesselSinus** | 0.222 | 0.095 | 0.006 | 0.006 | 0.203 | 0.203 | 0.246 | 0.246 | 0.095 | 0.045 |
| **Cortex-Vessel** | 0.267 | 0.082 | 0.007 | 0.007 | 0.256 | 0.256 | 0.283 | 0.283 | 0.101 | 0.055 |
| **Medulla-Vessel** | 0.223 | 0.073 | 0.009 | 0.009 | 0.188 | 0.188 | 0.239 | 0.239 | 0.136 | 0.059 |
| **Sinus-Vessel** | -0.012 | 0.035 | 0.007 | 0.007 | -0.021 | -0.021 | -0.010 | -0.010 | 0.009 | 0.070 |

**Supplementary Table 5**

Calculated biomechanical differences between cortex and medulla (Cortex-Medulla), cortex and sinus (Cortex-Sinus), medulla and sinus (Medulla-Sinus), cortex and vessel together with sinus (Cortex-VesselSinus), medulla and vessel together with sinus (Medulla-VesselSinus), cortex and vessel (Cortex-Vessel), medulla and vessel (Medulla-Vessel), and sinus and vessel (Sinus-Vessel).

|  |  |  |  | |  |  | |  | **Wilcoxon rank-sum tests** | |
| --- | --- | --- | --- | --- | --- | --- | --- | --- | --- | --- |
|  | **Hydration** | **ROI** | **median** | **SD** | **ROI** | **median** | **SD** | **Difference** | **paired p-value** | **non-paired p-value** |
| **Shear wave speed / m/s** | **h & d** | **Kidney** | **0.96** | **0.07** | **Cortex** | **1.01** | **0.08** | **-0.05** | **5.82E-11** | **1.34E-02** |
|  | **d** | **Kidney** | **0.95** | **0.07** | **Cortex** | **1.01** | **0.07** | **-0.06** | **1.53E-05** | **1.14E-01** |
|  | **h** | **Kidney** | **0.96** | **0.07** | **Cortex** | **1.02** | **0.09** | **-0.05** | **7.63E-06** | **6.37E-02** |
|  | **h & d** | **Kidney** | **0.96** | **0.07** | **Medulla** | **1.01** | **0.11** | **-0.05** | **5.85E-04** | **8.80E-02** |
|  | **d** | **Kidney** | **0.95** | **0.07** | **Medulla** | **1.01** | **0.11** | **-0.05** | **6.65E-02** | **3.08E-01** |
|  | **h** | **Kidney** | **0.96** | **0.07** | **Medulla** | **1.00** | **0.11** | **-0.04** | **4.75E-03** | **2.52E-01** |
|  | **h & d** | **Kidney** | **0.96** | **0.07** | **Sinus** | **0.77** | **0.06** | **0.19** | **5.82E-11** | **2.44E-16** |
|  | **d** | **Kidney** | **0.95** | **0.07** | **Sinus** | **0.79** | **0.06** | **0.16** | **1.53E-05** | **2.14E-08** |
|  | **h** | **Kidney** | **0.96** | **0.07** | **Sinus** | **0.76** | **0.05** | **0.21** | **7.63E-06** | **2.64E-09** |
|  | **h & d** | **Kidney** | **0.96** | **0.07** | **Vessel** | **0.77** | **0.06** | **0.18** | **2.91E-11** | **3.48E-15** |
|  | **d** | **Kidney** | **0.95** | **0.07** | **Vessel** | **0.77** | **0.06** | **0.18** | **7.63E-06** | **4.30E-08** |
|  | **h** | **Kidney** | **0.96** | **0.07** | **Vessel** | **0.77** | **0.05** | **0.19** | **7.63E-06** | **2.14E-08** |
|  | **h & d** | **Kidney** | **0.96** | **0.07** | **VesselSinus** | **0.77** | **0.05** | **0.18** | **2.91E-11** | **3.67E-16** |
|  | **d** | **Kidney** | **0.95** | **0.07** | **VesselSinus** | **0.80** | **0.06** | **0.16** | **7.63E-06** | **2.14E-08** |
|  | **h** | **Kidney** | **0.96** | **0.07** | **VesselSinus** | **0.77** | **0.05** | **0.19** | **7.63E-06** | **2.64E-09** |
|  | **h & d** | **Cortex** | **1.01** | **0.08** | **Medulla** | **1.01** | **0.11** | **0.00** | **3.71E-01** | **8.10E-01** |
|  | **d** | **Cortex** | **1.01** | **0.07** | **Medulla** | **1.01** | **0.11** | **0.00** | **8.32E-01** | **9.63E-01** |
|  | **h** | **Cortex** | **1.02** | **0.09** | **Medulla** | **1.00** | **0.11** | **0.01** | **3.25E-01** | **7.67E-01** |
|  | **h & d** | **Cortex** | **1.01** | **0.08** | **Sinus** | **0.77** | **0.06** | **0.24** | **2.91E-11** | **3.09E-18** |
|  | **d** | **Cortex** | **1.01** | **0.07** | **Sinus** | **0.79** | **0.06** | **0.21** | **7.63E-06** | **1.54E-09** |
|  | **h** | **Cortex** | **1.02** | **0.09** | **Sinus** | **0.76** | **0.05** | **0.26** | **7.63E-06** | **8.82E-10** |
|  | **h & d** | **Cortex** | **1.01** | **0.08** | **Vessel** | **0.77** | **0.06** | **0.24** | **2.91E-11** | **2.60E-17** |
|  | **d** | **Cortex** | **1.01** | **0.07** | **Vessel** | **0.77** | **0.06** | **0.24** | **7.63E-06** | **4.19E-09** |
|  | **h** | **Cortex** | **1.02** | **0.09** | **Vessel** | **0.77** | **0.05** | **0.24** | **7.63E-06** | **4.19E-09** |
|  | **h & d** | **Cortex** | **1.01** | **0.08** | **VesselSinus** | **0.77** | **0.05** | **0.24** | **2.91E-11** | **2.30E-18** |
|  | **d** | **Cortex** | **1.01** | **0.07** | **VesselSinus** | **0.80** | **0.06** | **0.21** | **7.63E-06** | **8.82E-10** |
|  | **h** | **Cortex** | **1.02** | **0.09** | **VesselSinus** | **0.77** | **0.05** | **0.25** | **7.63E-06** | **1.54E-09** |
|  | **h & d** | **Medulla** | **1.01** | **0.11** | **Sinus** | **0.77** | **0.06** | **0.24** | **5.82E-11** | **6.93E-15** |
|  | **d** | **Medulla** | **1.01** | **0.11** | **Sinus** | **0.79** | **0.06** | **0.21** | **1.53E-05** | **4.59E-07** |
|  | **h** | **Medulla** | **1.00** | **0.11** | **Sinus** | **0.76** | **0.05** | **0.25** | **7.63E-06** | **9.92E-09** |
|  | **h & d** | **Medulla** | **1.01** | **0.11** | **Vessel** | **0.77** | **0.06** | **0.23** | **2.91E-11** | **2.98E-14** |
|  | **d** | **Medulla** | **1.01** | **0.11** | **Vessel** | **0.77** | **0.06** | **0.23** | **7.63E-06** | **5.97E-07** |
|  | **h** | **Medulla** | **1.00** | **0.11** | **Vessel** | **0.77** | **0.05** | **0.23** | **7.63E-06** | **1.12E-07** |
|  | **h & d** | **Medulla** | **1.01** | **0.11** | **VesselSinus** | **0.77** | **0.05** | **0.23** | **2.91E-11** | **3.48E-15** |
|  | **d** | **Medulla** | **1.01** | **0.11** | **VesselSinus** | **0.80** | **0.06** | **0.21** | **7.63E-06** | **3.52E-07** |
|  | **h** | **Medulla** | **1.00** | **0.11** | **VesselSinus** | **0.77** | **0.05** | **0.23** | **7.63E-06** | **2.14E-08** |
|  | **h & d** | **Sinus** | **0.77** | **0.06** | **Vessel** | **0.77** | **0.06** | **0.00** | **1.87E-01** | **4.98E-01** |
|  | **d** | **Sinus** | **0.79** | **0.06** | **Vessel** | **0.77** | **0.06** | **0.02** | **7.99E-01** | **7.91E-01** |
|  | **h** | **Sinus** | **0.76** | **0.05** | **Vessel** | **0.77** | **0.05** | **-0.02** | **1.59E-02** | **1.71E-01** |
|  | **h & d** | **Sinus** | **0.77** | **0.06** | **VesselSinus** | **0.77** | **0.05** | **0.00** | **2.45E-01** | **7.44E-01** |
|  | **d** | **Sinus** | **0.79** | **0.06** | **VesselSinus** | **0.80** | **0.06** | **0.00** | **7.34E-01** | **1.00E+00** |
|  | **h** | **Sinus** | **0.76** | **0.05** | **VesselSinus** | **0.77** | **0.05** | **-0.01** | **9.34E-03** | **5.86E-01** |
|  | **h & d** | **Vessel** | **0.77** | **0.06** | **VesselSinus** | **0.77** | **0.05** | **0.00** | **1.84E-01** | **6.57E-01** |
|  | **d** | **Vessel** | **0.77** | **0.06** | **VesselSinus** | **0.80** | **0.06** | **-0.02** | **8.32E-01** | **8.64E-01** |
|  | **h** | **Vessel** | **0.77** | **0.05** | **VesselSinus** | **0.77** | **0.05** | **0.01** | **2.67E-02** | **3.57E-01** |
| **Alpha / 1/mm** | **h & d** | **Kidney** | **0.09** | **0.01** | **Cortex** | **0.09** | **0.01** | **0.00** | **3.96E-01** | **6.58E-01** |
|  | **d** | **Kidney** | **0.09** | **0.01** | **Cortex** | **0.09** | **0.01** | **0.00** | **5.80E-01** | **6.50E-01** |
|  | **h** | **Kidney** | **0.09** | **0.01** | **Cortex** | **0.09** | **0.01** | **0.00** | **5.80E-01** | **8.15E-01** |
|  | **h & d** | **Kidney** | **0.09** | **0.01** | **Medull** | **0.09** | **0.01** | **0.00** | **4.32E-01** | **4.84E-01** |
|  | **d** | **Kidney** | **0.09** | **0.01** | **Medull** | **0.09** | **0.01** | **0.00** | **4.17E-01** | **5.84E-01** |
|  | **h** | **Kidney** | **0.09** | **0.01** | **Medull** | **0.09** | **0.01** | **0.00** | **8.65E-01** | **6.28E-01** |
|  | **h & d** | **Kidney** | **0.09** | **0.01** | **Sinus** | **0.09** | **0.01** | **0.00** | **9.44E-01** | **6.50E-01** |
|  | **d** | **Kidney** | **0.09** | **0.01** | **Sinus** | **0.09** | **0.01** | **0.00** | **7.99E-01** | **3.72E-01** |
|  | **h** | **Kidney** | **0.09** | **0.01** | **Sinus** | **0.09** | **0.01** | **0.00** | **7.66E-01** | **7.91E-01** |
|  | **h & d** | **Kidney** | **0.09** | **0.01** | **Vessel** | **0.09** | **0.02** | **0.01** | **1.97E-01** | **6.87E-02** |
|  | **d** | **Kidney** | **0.09** | **0.01** | **Vessel** | **0.09** | **0.02** | **0.00** | **5.80E-01** | **1.00E+00** |
|  | **h** | **Kidney** | **0.09** | **0.01** | **Vessel** | **0.08** | **0.01** | **0.01** | **6.58E-03** | **9.63E-03** |
|  | **h & d** | **Kidney** | **0.09** | **0.01** | **VesselSinus** | **0.09** | **0.01** | **0.00** | **5.60E-01** | **4.91E-01** |
|  | **d** | **Kidney** | **0.09** | **0.01** | **VesselSinus** | **0.09** | **0.01** | **0.00** | **8.65E-01** | **4.81E-01** |
|  | **h** | **Kidney** | **0.09** | **0.01** | **VesselSinus** | **0.09** | **0.01** | **0.00** | **6.40E-01** | **7.19E-01** |
|  | **h & d** | **Cortex** | **0.09** | **0.01** | **Medulla** | **0.09** | **0.01** | **0.00** | **4.79E-01** | **3.26E-01** |
|  | **d** | **Cortex** | **0.09** | **0.01** | **Medulla** | **0.09** | **0.01** | **0.00** | **6.09E-01** | **5.01E-01** |
|  | **h** | **Cortex** | **0.09** | **0.01** | **Medulla** | **0.09** | **0.01** | **0.00** | **7.99E-01** | **5.01E-01** |
|  | **h & d** | **Cortex** | **0.09** | **0.01** | **Sinus** | **0.09** | **0.01** | **0.00** | **5.29E-01** | **5.87E-01** |
|  | **d** | **Cortex** | **0.09** | **0.01** | **Sinus** | **0.09** | **0.01** | **0.01** | **6.40E-01** | **3.55E-01** |
|  | **h** | **Cortex** | **0.09** | **0.01** | **Sinus** | **0.09** | **0.01** | **0.00** | **7.66E-01** | **8.64E-01** |
|  | **h & d** | **Cortex** | **0.09** | **0.01** | **Vessel** | **0.09** | **0.02** | **0.01** | **1.15E-01** | **6.21E-02** |
|  | **d** | **Cortex** | **0.09** | **0.01** | **Vessel** | **0.09** | **0.02** | **0.00** | **9.66E-01** | **9.63E-01** |
|  | **h** | **Cortex** | **0.09** | **0.01** | **Vessel** | **0.08** | **0.01** | **0.01** | **6.58E-03** | **1.29E-02** |
|  | **h & d** | **Cortex** | **0.09** | **0.01** | **VesselSinus** | **0.09** | **0.01** | **0.00** | **2.79E-01** | **3.43E-01** |
|  | **d** | **Cortex** | **0.09** | **0.01** | **VesselSinus** | **0.09** | **0.01** | **0.00** | **6.40E-01** | **3.72E-01** |
|  | **h** | **Cortex** | **0.09** | **0.01** | **VesselSinus** | **0.09** | **0.01** | **0.00** | **3.47E-01** | **6.06E-01** |
|  | **h & d** | **Medulla** | **0.09** | **0.01** | **Sinus** | **0.09** | **0.01** | **0.00** | **6.58E-01** | **7.84E-01** |
|  | **d** | **Medulla** | **0.09** | **0.01** | **Sinus** | **0.09** | **0.01** | **0.00** | **7.66E-01** | **9.38E-01** |
|  | **h** | **Medulla** | **0.09** | **0.01** | **Sinus** | **0.09** | **0.01** | **0.00** | **7.02E-01** | **7.19E-01** |
|  | **h & d** | **Medulla** | **0.09** | **0.01** | **Vessel** | **0.09** | **0.02** | **0.01** | **5.60E-01** | **2.69E-01** |
|  | **d** | **Medulla** | **0.09** | **0.01** | **Vessel** | **0.09** | **0.02** | **0.00** | **2.65E-01** | **7.19E-01** |
|  | **h** | **Medulla** | **0.09** | **0.01** | **Vessel** | **0.08** | **0.01** | **0.01** | **2.68E-02** | **5.91E-02** |
|  | **h & d** | **Medulla** | **0.09** | **0.01** | **VesselSinus** | **0.09** | **0.01** | **0.00** | **9.07E-01** | **9.42E-01** |
|  | **d** | **Medulla** | **0.09** | **0.01** | **VesselSinus** | **0.09** | **0.01** | **0.00** | **6.71E-01** | **9.13E-01** |
|  | **h** | **Medulla** | **0.09** | **0.01** | **VesselSinus** | **0.09** | **0.01** | **0.00** | **8.65E-01** | **8.39E-01** |
|  | **h & d** | **Sinus** | **0.09** | **0.01** | **Vessel** | **0.09** | **0.02** | **0.01** | **5.71E-01** | **2.55E-01** |
|  | **d** | **Sinus** | **0.09** | **0.01** | **Vessel** | **0.09** | **0.02** | **0.00** | **2.29E-01** | **5.42E-01** |
|  | **h** | **Sinus** | **0.09** | **0.01** | **Vessel** | **0.08** | **0.01** | **0.01** | **2.81E-03** | **2.90E-02** |
|  | **h & d** | **Sinus** | **0.09** | **0.01** | **VesselSinus** | **0.09** | **0.01** | **0.00** | **4.80E-01** | **7.30E-01** |
|  | **d** | **Sinus** | **0.09** | **0.01** | **VesselSinus** | **0.09** | **0.01** | **0.00** | **4.95E-01** | **8.88E-01** |
|  | **h** | **Sinus** | **0.09** | **0.01** | **VesselSinus** | **0.09** | **0.01** | **0.00** | **1.34E-02** | **3.90E-01** |
|  | **h & d** | **Vessel** | **0.09** | **0.02** | **VesselSinus** | **0.09** | **0.01** | **0.00** | **4.51E-01** | **3.32E-01** |
|  | **d** | **Vessel** | **0.09** | **0.02** | **VesselSinus** | **0.09** | **0.01** | **0.00** | **1.74E-01** | **5.63E-01** |
|  | **h** | **Vessel** | **0.08** | **0.01** | **VesselSinus** | **0.09** | **0.01** | **-0.01** | **2.81E-03** | **5.91E-02** |
| **Storage modulus / kPa** | **h & d** | **Kidney** | **0.62** | **0.08** | **Cortex** | **0.68** | **0.09** | **-0.06** | **2.56E-09** | **2.40E-02** |
|  | **d** | **Kidney** | **0.62** | **0.08** | **Cortex** | **0.69** | **0.09** | **-0.07** | **2.52E-04** | **1.52E-01** |
|  | **h** | **Kidney** | **0.62** | **0.08** | **Cortex** | **0.68** | **0.10** | **-0.06** | **7.63E-06** | **9.07E-02** |
|  | **h & d** | **Kidney** | **0.62** | **0.08** | **Medulla** | **0.68** | **0.13** | **-0.06** | **1.48E-04** | **5.45E-02** |
|  | **d** | **Kidney** | **0.62** | **0.08** | **Medulla** | **0.68** | **0.14** | **-0.06** | **2.37E-02** | **1.92E-01** |
|  | **h** | **Kidney** | **0.62** | **0.08** | **Medulla** | **0.68** | **0.12** | **-0.06** | **4.01E-03** | **1.52E-01** |
|  | **h & d** | **Kidney** | **0.62** | **0.08** | **Sinus** | **0.48** | **0.06** | **0.14** | **2.91E-11** | **6.39E-14** |
|  | **d** | **Kidney** | **0.62** | **0.08** | **Sinus** | **0.49** | **0.07** | **0.14** | **7.63E-06** | **1.60E-06** |
|  | **h** | **Kidney** | **0.62** | **0.08** | **Sinus** | **0.46** | **0.05** | **0.15** | **7.63E-06** | **9.92E-09** |
|  | **h & d** | **Kidney** | **0.62** | **0.08** | **Vessel** | **0.48** | **0.07** | **0.15** | **2.91E-11** | **1.94E-11** |
|  | **d** | **Kidney** | **0.62** | **0.08** | **Vessel** | **0.48** | **0.08** | **0.15** | **7.63E-06** | **9.10E-06** |
|  | **h** | **Kidney** | **0.62** | **0.08** | **Vessel** | **0.47** | **0.06** | **0.15** | **7.63E-06** | **1.26E-06** |
|  | **h & d** | **Kidney** | **0.62** | **0.08** | **VesselSinus** | **0.48** | **0.06** | **0.15** | **2.91E-11** | **1.78E-13** |
|  | **d** | **Kidney** | **0.62** | **0.08** | **VesselSinus** | **0.48** | **0.06** | **0.14** | **7.63E-06** | **1.60E-06** |
|  | **h** | **Kidney** | **0.62** | **0.08** | **VesselSinus** | **0.47** | **0.05** | **0.15** | **7.63E-06** | **2.14E-08** |
|  | **h & d** | **Cortex** | **0.68** | **0.09** | **Medulla** | **0.68** | **0.13** | **0.01** | **9.69E-01** | **9.51E-01** |
|  | **d** | **Cortex** | **0.69** | **0.09** | **Medulla** | **0.68** | **0.14** | **0.01** | **4.95E-01** | **9.13E-01** |
|  | **h** | **Cortex** | **0.68** | **0.10** | **Medulla** | **0.68** | **0.12** | **-0.01** | **5.23E-01** | **9.38E-01** |
|  | **h & d** | **Cortex** | **0.68** | **0.09** | **Sinus** | **0.48** | **0.06** | **0.20** | **2.91E-11** | **1.60E-16** |
|  | **d** | **Cortex** | **0.69** | **0.09** | **Sinus** | **0.49** | **0.07** | **0.20** | **7.63E-06** | **3.06E-08** |
|  | **h** | **Cortex** | **0.68** | **0.10** | **Sinus** | **0.46** | **0.05** | **0.21** | **7.63E-06** | **2.64E-09** |
|  | **h & d** | **Cortex** | **0.68** | **0.09** | **Vessel** | **0.48** | **0.07** | **0.21** | **2.91E-11** | **8.19E-15** |
|  | **d** | **Cortex** | **0.69** | **0.09** | **Vessel** | **0.48** | **0.08** | **0.21** | **7.63E-06** | **2.02E-07** |
|  | **h** | **Cortex** | **0.68** | **0.10** | **Vessel** | **0.47** | **0.06** | **0.21** | **7.63E-06** | **3.06E-08** |
|  | **h & d** | **Cortex** | **0.68** | **0.09** | **VesselSinus** | **0.48** | **0.06** | **0.21** | **2.91E-11** | **4.48E-16** |
|  | **d** | **Cortex** | **0.69** | **0.09** | **VesselSinus** | **0.48** | **0.06** | **0.21** | **7.63E-06** | **8.22E-08** |
|  | **h** | **Cortex** | **0.68** | **0.10** | **VesselSinus** | **0.47** | **0.05** | **0.21** | **7.63E-06** | **4.19E-09** |
|  | **h & d** | **Medulla** | **0.68** | **0.13** | **Sinus** | **0.48** | **0.06** | **0.20** | **2.04E-10** | **8.61E-12** |
|  | **d** | **Medulla** | **0.68** | **0.14** | **Sinus** | **0.49** | **0.07** | **0.19** | **3.81E-05** | **2.39E-05** |
|  | **h** | **Medulla** | **0.68** | **0.12** | **Sinus** | **0.46** | **0.05** | **0.22** | **7.63E-06** | **1.51E-07** |
|  | **h & d** | **Medulla** | **0.68** | **0.13** | **Vessel** | **0.48** | **0.07** | **0.20** | **1.46E-10** | **4.24E-11** |
|  | **d** | **Medulla** | **0.68** | **0.14** | **Vessel** | **0.48** | **0.08** | **0.20** | **1.53E-05** | **1.98E-05** |
|  | **h** | **Medulla** | **0.68** | **0.12** | **Vessel** | **0.47** | **0.06** | **0.21** | **7.63E-06** | **7.70E-07** |
|  | **h & d** | **Medulla** | **0.68** | **0.13** | **VesselSinus** | **0.48** | **0.06** | **0.20** | **8.73E-11** | **9.68E-12** |
|  | **d** | **Medulla** | **0.68** | **0.14** | **VesselSinus** | **0.48** | **0.06** | **0.20** | **2.29E-05** | **1.64E-05** |
|  | **h** | **Medulla** | **0.68** | **0.12** | **VesselSinus** | **0.47** | **0.05** | **0.22** | **7.63E-06** | **2.02E-07** |
|  | **h & d** | **Sinus** | **0.48** | **0.06** | **Vessel** | **0.48** | **0.07** | **0.01** | **1.52E-01** | **6.50E-01** |
|  | **d** | **Sinus** | **0.49** | **0.07** | **Vessel** | **0.48** | **0.08** | **0.01** | **8.32E-01** | **8.39E-01** |
|  | **h** | **Sinus** | **0.46** | **0.05** | **Vessel** | **0.47** | **0.06** | **0.00** | **2.08E-02** | **2.93E-01** |
|  | **h & d** | **Sinus** | **0.48** | **0.06** | **VesselSinus** | **0.48** | **0.06** | **0.01** | **3.28E-02** | **8.69E-01** |
|  | **d** | **Sinus** | **0.49** | **0.07** | **VesselSinus** | **0.48** | **0.06** | **0.01** | **5.28E-01** | **9.56E-01** |
|  | **h** | **Sinus** | **0.46** | **0.05** | **VesselSinus** | **0.47** | **0.05** | **0.00** | **6.58E-03** | **7.43E-01** |
|  | **h & d** | **Vessel** | **0.48** | **0.07** | **VesselSinus** | **0.48** | **0.06** | **0.00** | **2.70E-01** | **7.74E-01** |
|  | **d** | **Vessel** | **0.48** | **0.08** | **VesselSinus** | **0.48** | **0.06** | **0.00** | **6.70E-01** | **7.34E-01** |
|  | **h** | **Vessel** | **0.47** | **0.06** | **VesselSinus** | **0.47** | **0.05** | **0.00** | **3.42E-02** | **3.72E-01** |
| **Loss modulus / kPa** | **h & d** | **Kidney** | **0.41** | **0.06** | **Cortex** | **0.48** | **0.09** | **-0.07** | **2.91E-11** | **1.70E-03** |
|  | **d** | **Kidney** | **0.40** | **0.06** | **Cortex** | **0.45** | **0.09** | **-0.05** | **7.63E-06** | **4.02E-02** |
|  | **h** | **Kidney** | **0.43** | **0.06** | **Cortex** | **0.50** | **0.08** | **-0.07** | **7.63E-06** | **1.29E-02** |
|  | **h & d** | **Kidney** | **0.41** | **0.06** | **Medulla** | **0.46** | **0.11** | **-0.05** | **7.01E-03** | **1.62E-01** |
|  | **d** | **Kidney** | **0.40** | **0.06** | **Medulla** | **0.45** | **0.11** | **-0.05** | **1.81E-01** | **4.81E-01** |
|  | **h** | **Kidney** | **0.43** | **0.06** | **Medulla** | **0.46** | **0.10** | **-0.04** | **8.96E-03** | **2.79E-01** |
|  | **h & d** | **Kidney** | **0.41** | **0.06** | **Sinus** | **0.23** | **0.04** | **0.18** | **2.91E-11** | **1.23E-17** |
|  | **d** | **Kidney** | **0.40** | **0.06** | **Sinus** | **0.23** | **0.05** | **0.17** | **7.63E-06** | **8.82E-10** |
|  | **h** | **Kidney** | **0.43** | **0.06** | **Sinus** | **0.24** | **0.04** | **0.19** | **7.63E-06** | **2.64E-09** |
|  | **h & d** | **Kidney** | **0.41** | **0.06** | **Vessel** | **0.24** | **0.06** | **0.17** | **2.91E-11** | **5.85E-15** |
|  | **d** | **Kidney** | **0.40** | **0.06** | **Vessel** | **0.25** | **0.06** | **0.16** | **7.63E-06** | **3.06E-08** |
|  | **h** | **Kidney** | **0.43** | **0.06** | **Vessel** | **0.23** | **0.06** | **0.20** | **7.63E-06** | **8.22E-08** |
|  | **h & d** | **Kidney** | **0.41** | **0.06** | **VesselSinus** | **0.24** | **0.04** | **0.17** | **2.91E-11** | **2.04E-17** |
|  | **d** | **Kidney** | **0.40** | **0.06** | **VesselSinus** | **0.24** | **0.05** | **0.16** | **7.63E-06** | **2.64E-09** |
|  | **h** | **Kidney** | **0.43** | **0.06** | **VesselSinus** | **0.23** | **0.04** | **0.19** | **7.63E-06** | **4.19E-09** |
|  | **h & d** | **Cortex** | **0.48** | **0.09** | **Medulla** | **0.46** | **0.11** | **0.02** | **2.95E-02** | **2.11E-01** |
|  | **d** | **Cortex** | **0.45** | **0.09** | **Medulla** | **0.45** | **0.11** | **0.00** | **2.46E-01** | **4.24E-01** |
|  | **h** | **Cortex** | **0.50** | **0.08** | **Medulla** | **0.46** | **0.10** | **0.04** | **6.65E-02** | **2.39E-01** |
|  | **h & d** | **Cortex** | **0.48** | **0.09** | **Sinus** | **0.23** | **0.04** | **0.25** | **2.91E-11** | **1.81E-20** |
|  | **d** | **Cortex** | **0.45** | **0.09** | **Sinus** | **0.23** | **0.05** | **0.22** | **7.63E-06** | **2.20E-10** |
|  | **h** | **Cortex** | **0.50** | **0.08** | **Sinus** | **0.24** | **0.04** | **0.27** | **7.63E-06** | **4.41E-10** |
|  | **h & d** | **Cortex** | **0.48** | **0.09** | **Vessel** | **0.24** | **0.06** | **0.24** | **2.91E-11** | **9.43E-18** |
|  | **d** | **Cortex** | **0.45** | **0.09** | **Vessel** | **0.25** | **0.06** | **0.21** | **7.63E-06** | **8.82E-10** |
|  | **h** | **Cortex** | **0.50** | **0.08** | **Vessel** | **0.23** | **0.06** | **0.28** | **7.63E-06** | **4.19E-09** |
|  | **h & d** | **Cortex** | **0.48** | **0.09** | **VesselSinus** | **0.24** | **0.04** | **0.24** | **2.91E-11** | **8.59E-20** |
|  | **d** | **Cortex** | **0.45** | **0.09** | **VesselSinus** | **0.24** | **0.05** | **0.21** | **7.63E-06** | **4.41E-10** |
|  | **h** | **Cortex** | **0.50** | **0.08** | **VesselSinus** | **0.23** | **0.04** | **0.27** | **7.63E-06** | **8.82E-10** |
|  | **h & d** | **Medulla** | **0.46** | **0.11** | **Sinus** | **0.23** | **0.04** | **0.23** | **1.46E-10** | **1.41E-15** |
|  | **d** | **Medulla** | **0.45** | **0.11** | **Sinus** | **0.23** | **0.05** | **0.22** | **3.81E-05** | **3.52E-07** |
|  | **h** | **Medulla** | **0.46** | **0.10** | **Sinus** | **0.24** | **0.04** | **0.23** | **7.63E-06** | **2.64E-09** |
|  | **h & d** | **Medulla** | **0.46** | **0.11** | **Vessel** | **0.24** | **0.06** | **0.23** | **5.82E-11** | **2.17E-14** |
|  | **d** | **Medulla** | **0.45** | **0.11** | **Vessel** | **0.25** | **0.06** | **0.21** | **1.53E-05** | **7.70E-07** |
|  | **h** | **Medulla** | **0.46** | **0.10** | **Vessel** | **0.23** | **0.06** | **0.24** | **7.63E-06** | **3.06E-08** |
|  | **h & d** | **Medulla** | **0.46** | **0.11** | **VesselSinus** | **0.24** | **0.04** | **0.22** | **1.46E-10** | **6.63E-16** |
|  | **d** | **Medulla** | **0.45** | **0.11** | **VesselSinus** | **0.24** | **0.05** | **0.21** | **3.81E-05** | **1.51E-07** |
|  | **h** | **Medulla** | **0.46** | **0.10** | **VesselSinus** | **0.23** | **0.04** | **0.23** | **7.63E-06** | **2.64E-09** |
|  | **h & d** | **Sinus** | **0.23** | **0.04** | **Vessel** | **0.24** | **0.06** | **0.00** | **5.60E-01** | **9.15E-01** |
|  | **d** | **Sinus** | **0.23** | **0.05** | **Vessel** | **0.25** | **0.06** | **-0.01** | **5.80E-01** | **7.67E-01** |
|  | **h** | **Sinus** | **0.24** | **0.04** | **Vessel** | **0.23** | **0.06** | **0.01** | **8.32E-01** | **6.96E-01** |
|  | **h & d** | **Sinus** | **0.23** | **0.04** | **VesselSinus** | **0.24** | **0.04** | **-0.01** | **5.65E-01** | **8.43E-01** |
|  | **d** | **Sinus** | **0.23** | **0.05** | **VesselSinus** | **0.24** | **0.05** | **-0.01** | **5.79E-01** | **7.08E-01** |
|  | **h** | **Sinus** | **0.24** | **0.04** | **VesselSinus** | **0.23** | **0.04** | **0.00** | **8.32E-01** | **8.15E-01** |
|  | **h & d** | **Vessel** | **0.24** | **0.06** | **VesselSinus** | **0.24** | **0.04** | **0.00** | **4.91E-01** | **7.53E-01** |
|  | **d** | **Vessel** | **0.25** | **0.06** | **VesselSinus** | **0.24** | **0.05** | **0.01** | **3.29E-01** | **9.19E-01** |
|  | **h** | **Vessel** | **0.23** | **0.06** | **VesselSinus** | **0.23** | **0.04** | **-0.01** | **7.99E-01** | **5.01E-01** |
| **Phase-angle / rad * 2/pi** | **h & d** | **Kidney** | **0.36** | **0.02** | **Cortex** | **0.39** | **0.03** | **-0.02** | **6.41E-06** | **2.95E-03** |
|  | **d** | **Kidney** | **0.36** | **0.02** | **Cortex** | **0.38** | **0.03** | **-0.02** | **1.78E-03** | **6.19E-02** |
|  | **h** | **Kidney** | **0.36** | **0.02** | **Cortex** | **0.39** | **0.03** | **-0.03** | **3.28E-04** | **1.06E-02** |
|  | **h & d** | **Kidney** | **0.36** | **0.02** | **Medull** | **0.37** | **0.04** | **-0.01** | **1.08E-01** | **1.72E-01** |
|  | **d** | **Kidney** | **0.36** | **0.02** | **Medull** | **0.36** | **0.04** | **0.00** | **5.23E-01** | **4.81E-01** |
|  | **h** | **Kidney** | **0.36** | **0.02** | **Medull** | **0.38** | **0.04** | **-0.02** | **1.54E-01** | **2.14E-01** |
|  | **h & d** | **Kidney** | **0.36** | **0.02** | **Sinus** | **0.29** | **0.04** | **0.07** | **2.21E-07** | **2.54E-10** |
|  | **d** | **Kidney** | **0.36** | **0.02** | **Sinus** | **0.29** | **0.05** | **0.07** | **1.05E-03** | **9.10E-06** |
|  | **h** | **Kidney** | **0.36** | **0.02** | **Sinus** | **0.30** | **0.04** | **0.06** | **7.63E-05** | **1.64E-05** |
|  | **h & d** | **Kidney** | **0.36** | **0.02** | **Vessel** | **0.30** | **0.05** | **0.07** | **5.06E-08** | **3.31E-08** |
|  | **d** | **Kidney** | **0.36** | **0.02** | **Vessel** | **0.30** | **0.05** | **0.06** | **6.71E-04** | **9.86E-04** |
|  | **h** | **Kidney** | **0.36** | **0.02** | **Vessel** | **0.29** | **0.05** | **0.08** | **7.63E-05** | **7.42E-06** |
|  | **h & d** | **Kidney** | **0.36** | **0.02** | **VesselSinus** | **0.30** | **0.03** | **0.07** | **2.91E-10** | **5.34E-12** |
|  | **d** | **Kidney** | **0.36** | **0.02** | **VesselSinus** | **0.29** | **0.03** | **0.07** | **2.29E-05** | **3.94E-06** |
|  | **h** | **Kidney** | **0.36** | **0.02** | **VesselSinus** | **0.30** | **0.03** | **0.07** | **1.53E-05** | **2.02E-06** |
|  | **h & d** | **Cortex** | **0.39** | **0.03** | **Medulla** | **0.37** | **0.04** | **0.02** | **2.20E-01** | **3.26E-01** |
|  | **d** | **Cortex** | **0.38** | **0.03** | **Medulla** | **0.36** | **0.04** | **0.02** | **5.23E-01** | **5.42E-01** |
|  | **h** | **Cortex** | **0.39** | **0.03** | **Medulla** | **0.38** | **0.04** | **0.01** | **4.68E-01** | **4.24E-01** |
|  | **h & d** | **Cortex** | **0.39** | **0.03** | **Sinus** | **0.29** | **0.04** | **0.09** | **3.67E-08** | **1.75E-12** |
|  | **d** | **Cortex** | **0.38** | **0.03** | **Sinus** | **0.29** | **0.05** | **0.10** | **3.28E-04** | **2.53E-06** |
|  | **h** | **Cortex** | **0.39** | **0.03** | **Sinus** | **0.30** | **0.04** | **0.09** | **3.81E-05** | **1.51E-07** |
|  | **h & d** | **Cortex** | **0.39** | **0.03** | **Vessel** | **0.30** | **0.05** | **0.09** | **5.92E-08** | **4.73E-11** |
|  | **d** | **Cortex** | **0.38** | **0.03** | **Vessel** | **0.30** | **0.05** | **0.08** | **6.71E-04** | **1.55E-04** |
|  | **h** | **Cortex** | **0.39** | **0.03** | **Vessel** | **0.29** | **0.05** | **0.10** | **5.34E-05** | **1.12E-07** |
|  | **h & d** | **Cortex** | **0.39** | **0.03** | **VesselSinus** | **0.30** | **0.03** | **0.09** | **4.07E-10** | **2.55E-14** |
|  | **d** | **Cortex** | **0.38** | **0.03** | **VesselSinus** | **0.29** | **0.03** | **0.09** | **1.53E-05** | **7.70E-07** |
|  | **h** | **Cortex** | **0.39** | **0.03** | **VesselSinus** | **0.30** | **0.03** | **0.09** | **3.81E-05** | **2.14E-08** |
|  | **h & d** | **Medulla** | **0.37** | **0.04** | **Sinus** | **0.29** | **0.04** | **0.08** | **8.04E-07** | **1.60E-09** |
|  | **d** | **Medulla** | **0.36** | **0.04** | **Sinus** | **0.29** | **0.05** | **0.08** | **8.39E-04** | **2.39E-05** |
|  | **h** | **Medulla** | **0.38** | **0.04** | **Sinus** | **0.30** | **0.04** | **0.08** | **4.20E-04** | **1.64E-05** |
|  | **h & d** | **Medulla** | **0.37** | **0.04** | **Vessel** | **0.30** | **0.05** | **0.07** | **9.35E-08** | **1.23E-08** |
|  | **d** | **Medulla** | **0.36** | **0.04** | **Vessel** | **0.30** | **0.05** | **0.06** | **8.39E-04** | **1.45E-03** |
|  | **h** | **Medulla** | **0.38** | **0.04** | **Vessel** | **0.29** | **0.05** | **0.09** | **7.63E-05** | **2.02E-06** |
|  | **h & d** | **Medulla** | **0.37** | **0.04** | **VesselSinus** | **0.30** | **0.03** | **0.07** | **7.36E-09** | **8.11E-11** |
|  | **d** | **Medulla** | **0.36** | **0.04** | **VesselSinus** | **0.29** | **0.03** | **0.07** | **7.63E-05** | **1.64E-05** |
|  | **h** | **Medulla** | **0.38** | **0.04** | **VesselSinus** | **0.30** | **0.03** | **0.08** | **5.34E-05** | **3.17E-06** |
|  | **h & d** | **Sinus** | **0.29** | **0.04** | **Vessel** | **0.30** | **0.05** | **0.00** | **8.83E-01** | **8.19E-01** |
|  | **d** | **Sinus** | **0.29** | **0.05** | **Vessel** | **0.30** | **0.05** | **-0.01** | **3.04E-01** | **2.52E-01** |
|  | **h** | **Sinus** | **0.30** | **0.04** | **Vessel** | **0.29** | **0.05** | **0.01** | **1.81E-01** | **1.81E-01** |
|  | **h & d** | **Sinus** | **0.29** | **0.04** | **VesselSinus** | **0.30** | **0.03** | **0.00** | **8.30E-01** | **7.40E-01** |
|  | **d** | **Sinus** | **0.29** | **0.05** | **VesselSinus** | **0.29** | **0.03** | **0.00** | **1.85E-01** | **4.67E-01** |
|  | **h** | **Sinus** | **0.30** | **0.04** | **VesselSinus** | **0.30** | **0.03** | **0.00** | **3.32E-02** | **6.81E-01** |
|  | **h & d** | **Vessel** | **0.30** | **0.05** | **VesselSinus** | **0.30** | **0.03** | **0.00** | **8.63E-01** | **6.52E-01** |
|  | **d** | **Vessel** | **0.30** | **0.05** | **VesselSinus** | **0.29** | **0.03** | **0.01** | **3.94E-01** | **5.27E-01** |
|  | **h** | **Vessel** | **0.29** | **0.05** | **VesselSinus** | **0.30** | **0.03** | **-0.01** | **2.84E-01** | **2.93E-01** |

**Supplementary Table 6** Biomechanical differences between all renal regions of interest (ROI). “VesselSinus” summarized the ROI of the Sinus and Vessel. Significant p-values are highlighted in grey. Wilcoxon rank-sum tests, two-sided, paired and non-paired, and case 8 excluded.
