# Supplementary figures and images for: Magnetic resonance elastography resolving all gross anatomical segments of the kidney during controlled hydration
Source: Front Physiol. 2024 Feb 7;15:1327407. doi: 10.3389/fphys.2024.1327407 (PMC10880033; doi:10.3389/fphys.2024.1327407)

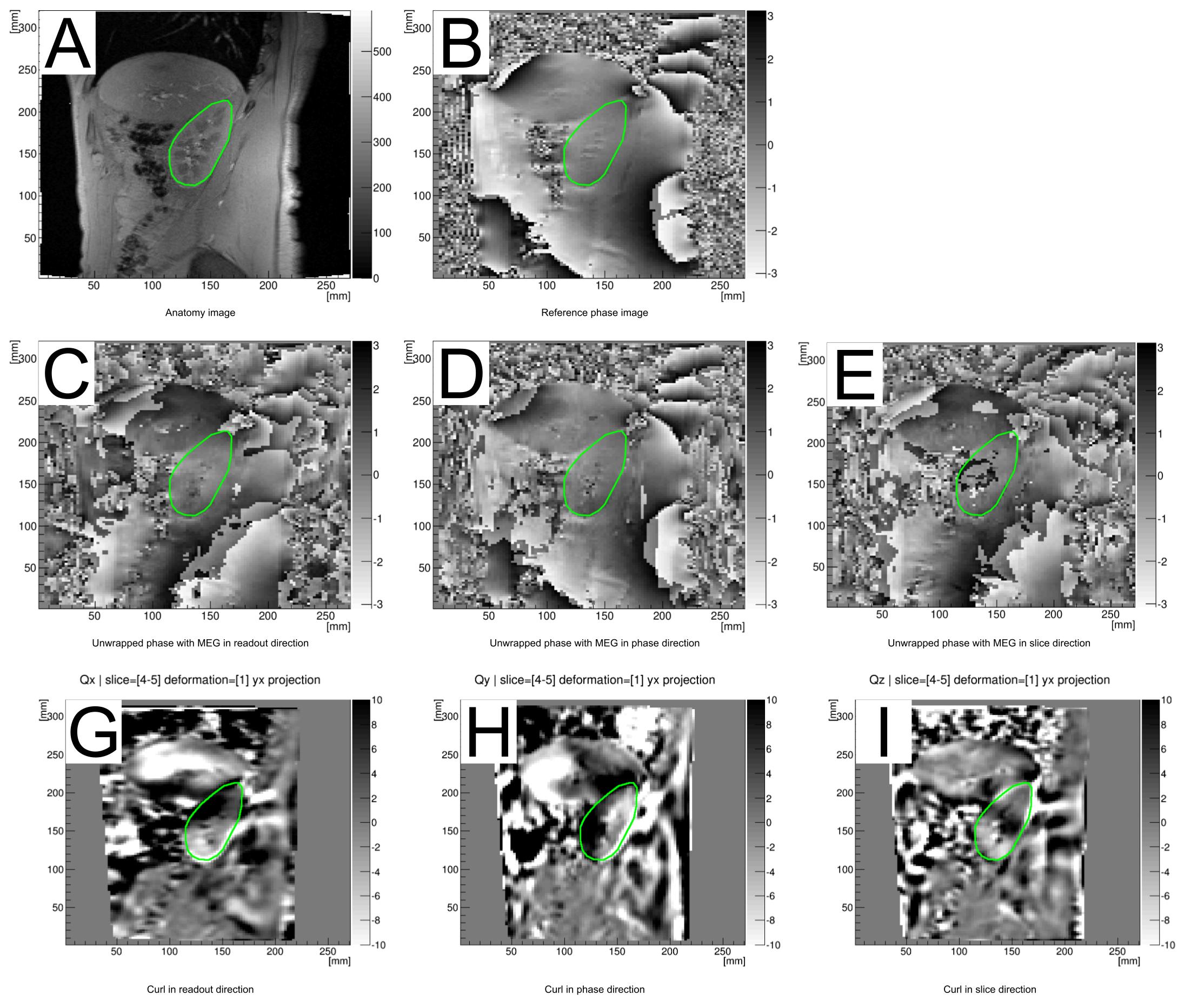

Supplement: Supplementary file 2 [file Image1.JPEG]

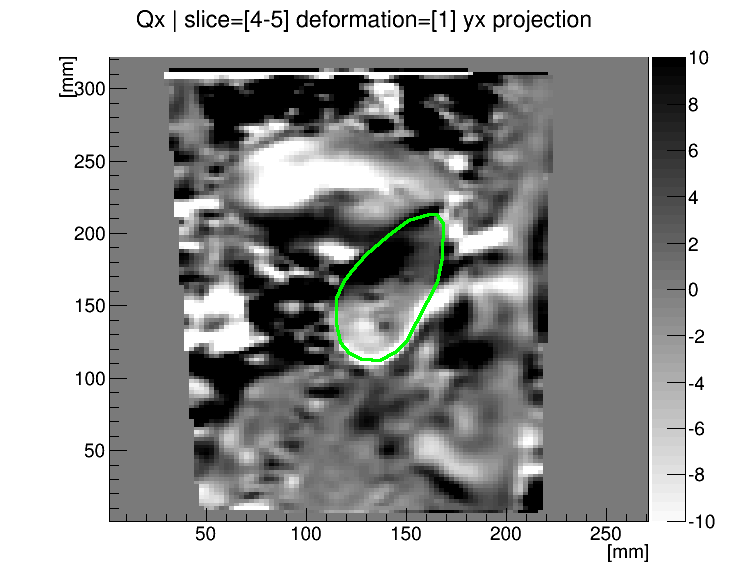

Supplement: Supplementary file 3 [file DataSheet1.zip › Supplementary Data Sheet 1/Qx.gif]

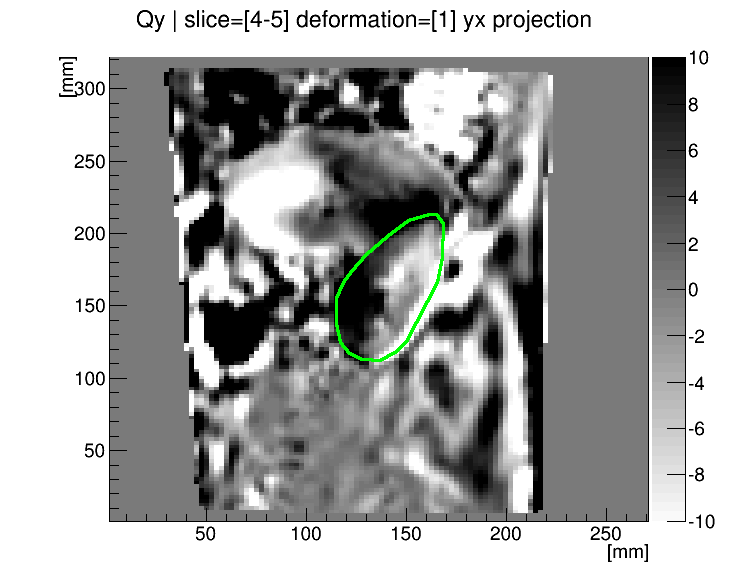

Supplement: Supplementary file 3 [file DataSheet1.zip › Supplementary Data Sheet 1/Qy.gif]

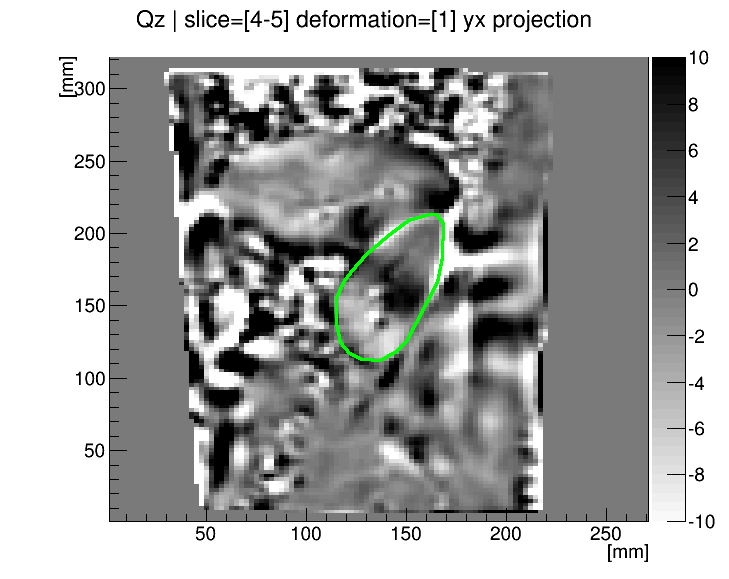

Supplement: Supplementary file 3 [file DataSheet1.zip › Supplementary Data Sheet 1/Qz.gif]
